# *Bacillus subtilis* and *Macleaya cordata* extract regulate the rumen microbiota associated with enteric methane emission in dairy cows

**DOI:** 10.1186/s40168-023-01654-3

**Published:** 2023-10-19

**Authors:** Peng Jia, Li-feng Dong, Yan Tu, Qi-yu Diao

**Affiliations:** 1 Institute of Feed Research, Chinese Academy of Agricultural Sciences/Sino-US Joint Lab On Nutrition and Metabolism of Ruminant/Key Laboratory of Feed Biotechnology of the Ministry of Agriculture and Rural Affairs, Beijing, 100081 People’s Republic of China; 2 State Key Laboratory of Grassland Agro-Ecosystems, Key Laboratory of Grassland Livestock Industry Innovation, Ministry of Agriculture and Rural Affairs, College of Pastoral Agriculture Science and Technology, Lanzhou University, Lanzhou, 730020 People’s Republic of China

**Keywords:** *Bacillus subtilis*, Dairy cows, *Macleaya cordata* extract, Metagenomics, Methane, Rumen microbiota

## Abstract

**Background:**

Ruminant livestock production is a considerable source of enteric methane (CH_4_) emissions. In a previous study, we found that dietary inclusions of *Bacillus subtilis* (BS) and *Macleaya cordata* extract (MCE) increased dry matter intake and milk production, while reduced enteric CH_4_ emission in dairy cows. The objective of this study was to further elucidate the impact of feeding BS and MCE on rumen methanogenesis in dairy cows using rumen metagenomics techniques.

**Results:**

Sixty dairy cows were blocked in 20 groups of 3 cows accordingly to their live weight, milk yield, and days in milk, and within each group, the 3 cows were randomly allocated to 1 of 3 treatments: control diet (CON), control diet plus BS (BS), and control diet plus MCE (MCE). After 75 days of feeding experimental diets, 12 cows were selected from each treatment for collection of rumen samples for the metagenomic sequencing. Results showed that BS decreased ruminal acetate and butyrate, while increased propionate concentrations, resulting in decreased acetate:propionate ratio. The metagenomics analysis revealed that MCE reduced relative abundances of *Methanobrevibacter wolinii, Methanobrevibacter sp. AbM4, Candidatus Methanomassiliicoccus intestinalis, Methanobrevibacter cuticularis, Methanomicrobium mobile, Methanobacterium formicicum,* and *Methanobacterium congolense*. Both BS and MCE reduced relative abundances of *Methanosphaera sp*. *WGK6* and *Methanosphaera stadtmanae*. The co-occurrence network analysis of rumen bacteria and archaea revealed that dietary treatments influenced microbial interaction patterns, with BS and MCE cows having more and stronger associations than CON cows. The random forest and heatmaps analysis demonstrated that the *Halopenitus persicus* was positively correlated with fat- and protein-corrected milk yield; *Clostridium sp*. *CAG 269*, *Clostridium sp. 27 14*, *Haloarcula rubripromontorii*, and *Methanobrevibacter curvatus* were negatively correlated with rumen acetate and butyrate concentrations, and acetate:propionate ratio, whereas *Selenomonas rumiantium* was positively correlated with those variables.

**Conclusions:**

The present results provided new information for mitigation of enteric methane emissions of dairy cows by feeding BS and MCE to influence rumen microbial activities. This fundamental knowledge is essential for developing enteric CH4 reduction strategies to mitigate climate change and reduce dietary energy waste.

Video Abstract

**Supplementary Information:**

The online version contains supplementary material available at 10.1186/s40168-023-01654-3.

## Background

Anthropogenic greenhouse gas emissions have already raised global temperatures by a mean of 1°C above pre-industrial levels, with annual emissions continuing to rise, so slowing global warming is a huge global challenge [1]. Sectors including agriculture, forestry, and other land use contribute greatly to greenhouse gas emissions, accounting for 22% of global emissions [1]. Methane (CH_4_) is more than 25 times more capable of trapping heat in the atmosphere than carbon dioxide (CO_2_), but has a lifetime in the atmosphere 1/5 to 1/20 that of CO_2_ [2]. This suggests that reducing CH_4_ emissions is the key to mitigate climate change in a short term. To date, CH_4_ emissions from livestock production account for about 12% of anthropogenic warming [3], as livestock production systems emit nearly 80% of the CH_4_ from agriculture, with enteric fermentation alone producing 87–97 Tg of CH_4_ per year [4]. Therefore, the mitigation of CH_4_ emissions from ruminants is critical for the achievement of the Paris Agreement’s temperature targets.

Dairy cattle and other ruminants play a major role in the sustainability of global agricultural systems. They use microorganisms in the rumen to convert feed resources unsuitable for human consumption into meat and dairy products. In particular, cows produce 80% of the milk in the global human food supply chain [5]. Over the past century, the global cattle population has tripled to meet the demand for animal protein [6]. Under current policies, agricultural CH_4_ emissions are projected to increase by about 30% in 2050 from 2010 levels [7]. Dairy industry faces serious challenges in the implementation of these environmental regulations. Mitigating enteric CH_4_ emissions can also reduce energy waste and thus improves dietary energy utilization efficiency in ruminants.

Feed additives have been widely used to manipulate rumen microbial activity for mitigation of enteric CH_4_ emissions in the ruminant livestock industry. *Bacillus subtilis* (BS) is a gram-positive bacterium, which can form cold- and heat-resistant spores and has metabolic activity of producing extracellular enzymes [8]. Studies showed that BS had probiotic ability and lignin-degradation capacity and feeding BS could increase milk yield, while decreased the ruminal acetate to propionate ratio [9, 10]. Sanguinarine and chelerythrine are the key bioactive compositions of the extract obtained from the *Macleaya cordata*, which is a perennial herb of traditional Chinese medicine [11]. *Macleaya cordata* extract (MCE) has the immune boosting capability, as well as antioxidant, antibacterial, anti-inflammatory, and anti-tumor activities [12]. Our previous study demonstrated that feeding BS and MCE increased milk production and reduced CH_4_ emissions per kilogram of fat-corrected or energy-corrected milk in dairy cows [13].

How to abate CH_4_ emissions from ruminants to cope with the increasing atmospheric concentrations of CH_4_ depends on our knowledge of the mechanisms of methanogenesis. Deciphering the details of methanogenic mechanisms to propose enteric CH_4_ mitigation strategies is a subject of growing international concern. Complex rumen microbes enable ruminants to digest fibrous feed through microbial-mediated fermentation; however, this process also inevitably produces CH_4_. Understanding of the rumen ecosystem can help scientists manipulate rumen microbial activity for the development of sustainable dairy production systems. The metagenomics technique is a culture-independent method that enables the assembly of near-complete microbial genomes directly from metagenomic sequencing data [14]. The metagenomics analysis can help scientists reveal the variations among rumen microbial communities and identify their functions and interactions, which has been successfully applied to different ecosystems [15]. The current study aimed to use the metagenomics analysis to evaluate the impact of feeding BS and NCE on microbiome-dependent mechanisms for mitigation of methanogenesis in the rumen of dairy cows.

## Methods

### Cows and experimental design

The experiment was conducted at the Yinxiangweiye International Third Farm (Cao County, Shandong Province, CHN). The cows involved in the experiment were managed and cared for according to the protocols approved by the Institute of Feed Research of Chinese Academy of Agricultural Sciences. The experiment was conducted with 60 multiparous lactating Holstein cows averaging (± SD) day in milk 145 ± 12.5 days, milk yield 38.3 ± 3.3 kg/day, and parity 2.5 ± 0.9. Treatments were as follows: (1) control (CON), (2) control diet supplemented with BS at 50 g/head/day (BS), or (3) control diet supplemented with MCE at 450 mg/head/day (MCE). The control diet was formulated to yield a 43:57 forage-to-concentrate ratio (dry matter basis, Table S1) and offered as total mixed ration. The BS (2 × 10^10^ CFU/g) and MCE (i.e., 40% sanguinarine and 20% chelerythrine) were fed to each cow individually during morning feeding every day [13]. Cows were randomly assigned to a treatment, and cows of each treatment were housed in a freestall barn (30 m × 12 m). Experimental period consisted of 15 days of adaptation and 60 days of measurements [13].

### Sampling and measurements

The measurements of milk production, milk composition, and enteric CH_4_ emissions were previously described [13]. Rumen samples were collected for each cow using an oral stomach tube [16], 2 h after morning feeding on day 75. Rumen contents were analyzed immediately for pH (basic pH meter PB-20, Startorius AG, Germany). Two 1-mL rumen content samples were transferred to a microfuge tube for metagenome analysis and stored at − 80°C. Then, the rumen contents were filtered through four layers of cheesecloth. A 10-mL aliquot was transferred to a tube for ruminal volatile fatty acid (VFA) analysis [17]; another 10-mL ruminal fluid sample was transferred to another tube for ruminal ammonia nitrogen (NH_3_-N) analysis [18]. These two samples were stored at − 20°C until further analysis.

### DNA extraction, metagenome sequencing, and data processing

Twelve cows were randomly selected from each group for further metagenomic analysis. Total genomic DNA was extracted from each rumen content sample based on modified repeated bead-beating plus column method [19]. DNA integrity was controlled by agarose gel electrophoresis. DNA quality and concentration were evaluated using a NanoDrop 2000 spectrophotometer (Thermo Fisher Scientific, Waltham, MA, USA). Construction of individual metagenome libraries was performed using TrueSeq DNA PCR-Free Library Preparation Kit (Illumina, San Diego, CA, USA). The metagenome libraries were sequenced (2 × 150 paired-end) in Beijing Allwegene Tech Ltd. (Beijing, China) using the Illumina HiSeq 3000 platform.

The quality control of each metagenomic sequence reads was performed using Sickle (version 1.33, https://github.com/najoshi/sickle). The quality-filtered reads were aligned to the bovine genome using BWA (http://bio-bwa.sourceforge.net) to filter out host DNA [20]. The remaining reads were de novo assembled for each sample using Megahit v1.1.2 (https://github.com/voutcn/megahit) [21]. Open reading frames (ORFs) were predicted from the assembled contigs with the length > 300 bp using MetaGene [22]. Assembled contigs were then clustered and non-redundancies were identified using CD-HI with 95% cutoff sequencing identity [23]. Original sequencing reads were mapped to predicted genes to estimate their abundances using SOAPaligner [24].

The rumen microbiota was taxonomically assigned using DIAMOND [25] against the RefSeq database [26]. Taxonomic profiles were summarized at domain, phylum, genus, and species levels, with relative abundances at those taxonomic ranks calculated. Microbial taxa with a relative abundance > 0.1% in at least 50% of cows within each group were used for downstream analysis. Contigs were annotated using DIAMOND against the KEGG database (http://www.genome.jp/kegg/) with an *E* value of 1e-5. The CAZy annotation was performed using USEARCH (http://www.drive5.com/usearch/). Abundances of KEGG Orthology (KO), pathway, KEGG enzyme, Module, and CAZymes were normalized into counts per million reads (cpm) for downstream analysis. The KEGG modules, pathways, KEGG enzymes, and CAZymes with cpm > 5 in at least 50% of animals within each group were used for the downstream analysis.

### Bioinformatics and statistical analysis

Co-occurrence among the bacterial taxa or archaeal taxa was analyzed using the SparCC program with the default settings. Spearman correlation analysis was performed to associate microbial taxa with functions. Only the species-level bacterial taxa or archaeal taxa with a relative abundance > 0.1% were used in the co-occurrence and correlation analysis, and only those with a correlation coefficient of > 0.6 or <  − 0.6 and a *P* value of < 0.05 were used in cooccurrence network analysis. The Cytoscape (Version 3.2.1, http://www.cytoscape.org.) was used to visualize networks. The “CytoHubba” function in Cytoscape software based on Maximal Clique Centrality method was used to calculate the hubs of microorganisms in the networks.

The randomForest package in R was used for random forest analysis, and rumen microorganisms were used as input of the random forest model. Rank the importance of microorganisms in the model according to mean decrease accuracy score. The UC-RF algorithm was used to determine the best predictive microorganisms based on the maximum area under the curve. The model was further evaluated by applying a 99-fold cross-validation scheme using the rfUtilities package in R (Version2.1–5, https:// cran.r-project.org/web/packages/rfUtilities/index.html). The top three microbiomes were selected to make a correlation heat map with cow phenotypes and rumen fermentation characteristics.

### Statistical analysis

Cow phenotypes and rumen fermentation characteristics were compared using one-way ANOVA procedure of SAS version 9.2. Rumen microbial domains, phyla, genera, and species were compared using Kruskal–Wallis multiple comparisons test, with the *P* value < 0.05 being considered as significantly different. The abundances of microbial metabolic pathways, modules, KEGG enzymes, and CAZymes were also compared among three groups using Kruskal–Wallis multiple comparisons test, and significant differences were considered by a *P* value < 0.05. The SPSS 19.0 was used to complete the Spearman rank correlation coefficients and significance tests between rumen microbial taxa, with the *P* value < 0.05 being considered as significantly different.

## Results

### Cow phenotypes, rumen fermentation characteristics

BS and MCE in the diet increased (*P* < 0.01) fat- and protein-corrected milk (FPCM) yield and decreased (*P* < 0.05) CH_4_/FPCM compared with CON (Table 1). The concentration of NH_3_-N in rumen was increased (*P* < 0.05) by the MCE diet, relative to the BS diet. We detected no effect (*P* > 0.05) of treatments on rumen pH value and total VFA concentration. However, acetate:propionate ratio was reduced (*P* < 0.01) by the BS diet, due to decreased (*P* < 0.01) the molar proportion of the acetate and increased (*P* < 0.01) the molar proportion of the propionate, compared with CON. Compared with CON and MCE, BS reduced (*P* < 0.05) the molar proportion of the butyrate.

**Table 1:** Comparison of physiological parameters and rumen fermentation characteristics among CON, BS, and MCE cows (*n* = 60)

Item	Dietary treatment			SEM	*P* value
	CON	BS	MCE		
DMI, kg/day	22.78^b^	25.96^a^	25.35^a^	0.210	0.040
FPCM, kg/day	30.33^b^	33.35^a^	32.62^a^	0.271	< 0.001
CH_4_/FPCM, g/kg	13.22^a^	12.40^b^	12.45^b^	0.150	0.038
Rumen pH	6.22	6.04	6.18	0.040	0.163
Total VFA, mmol/L	109.94	112.18	108.58	1.881	0.743
Acetate, %	59.79^a^	57.13^b^	58.72^ab^	0.360	0.008
Propionate, %	25.43^b^	29.28^a^	26.81^b^	0.442	0.001
Butyrate, %	11.84^a^	10.89^b^	11.85^a^	0.182	0.045
Isobutyrate, %	0.67	0.48	0.59	0.044	0.217
Valerate, %	1.37	1.34	1.22	0.030	0.074
Isovalerate, %	0.89	0.89	0.81	0.035	0.561
Ammonia N, mg/dL	11.6^ab^	10.99^b^	12.34^a^	0.229	0.049
Acetate/propionate	2.41^a^	1.98^b^	2.21^a^	0.051	0.002

Data were analyzed using the one-way ANOVA procedure (*n* = 20 per group). ^a,b^Means bearing different superscripts in the same row differ significantly (*P* value < 0.05)

*DMI* dry matter intake, *FPCM* fat- and protein-corrected milk (kg/d) = milk (kg) × [0.337 + 0.116 × fat (%) + 0.06 × protein (%)] [27]. CH_4_, methane. SEM, standard error of the mean, *CON* control diet, *BS* control diet plus *Bacillus subtilis*, *MCE* control diet plus *Macleaya cordata* extract

### Profiling of the rumen metagenome

Metagenome sequencing generated a total of 1,528,562,031 reads, with 43,673,201 ± 622,514 reads (mean ± standard error of the mean) per sample. Furthermore, a total of 1,523,315,546 reads were retained, with 43,523,301 ± 619,687 per sample (Table S2).

**Fig. 1:**
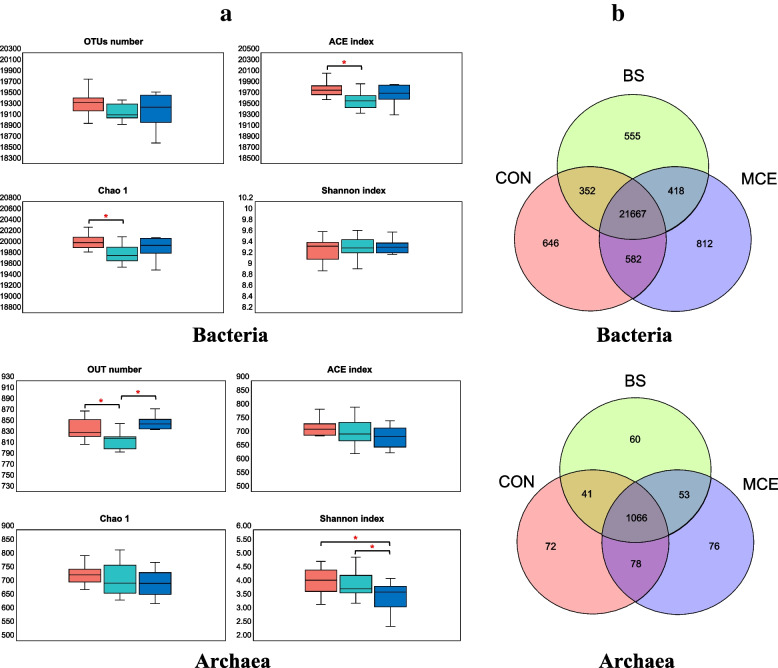
The diversity of rumen microbial communities in cows. **a** Optional taxonomic unit (OTU) number and α-diversity indexes. **b** Venn diagram of OTUs in three groups. The difference among three groups was identified by Kruskal–Wallis multiple comparisons, and asterisk indicated the significant difference (*P* value < 0.05). CON control diet, BS control diet plus *Bacillus subtilis*, MCE control diet plus *Macleaya cordata* extract

The rumen microbiomes of the three groups were compared in terms of microbial domains. The Eukaryota were remarkably different between the MCE and BS groups (*P* < 0.05, Table S3). In terms of bacterial α-diversity, BS had a lower ACE (*P* < 0.05) and Chao (*P* < 0.05) indexes compared to the CON group, but there were no differences (*P* > 0.05) in these measures between the CON and MCE groups (Fig. 1a). In terms of α-diversity in archaea, the OTU number of BS was lower (*P* < 0.05) than that of the CON and MCE groups and MCE had a lower Shannon index (*P* < 0.05) compared to the CON and BS groups (Fig. 1a). Venn diagram of bacteria showed that at the OTU level, there were 21,667 common core OTUs in each group; 646, 555, and 812 unique OTUs in the CON, BS, and MCE groups, respectively (Fig. 1b). In addition, Venn diagram of archaea showed that at the OTU level, there were 1066 common core OTUs in each group; 72, 60, and 76 unique OTUs in the CON, BS, and MCE groups, respectively (Fig. 1b).

Enteric CH_4_ emission from cows is exclusively produced by archaea. Bacteria are the predominant components of total rumen microbiota, and bacterial fermentation provides substrates such as H_2_, CO_2_, VFA, and methyl compounds for methanogenic archaea to synthesize CH_4_ [28]. Therefore, only bacteria and archaea were considered in the downstream comparison of rumen microbial taxa among the three groups of cows.

### Compositional profiles of the rumen microbiome and taxonomic differences among cows in three groups

**Fig. 2:**
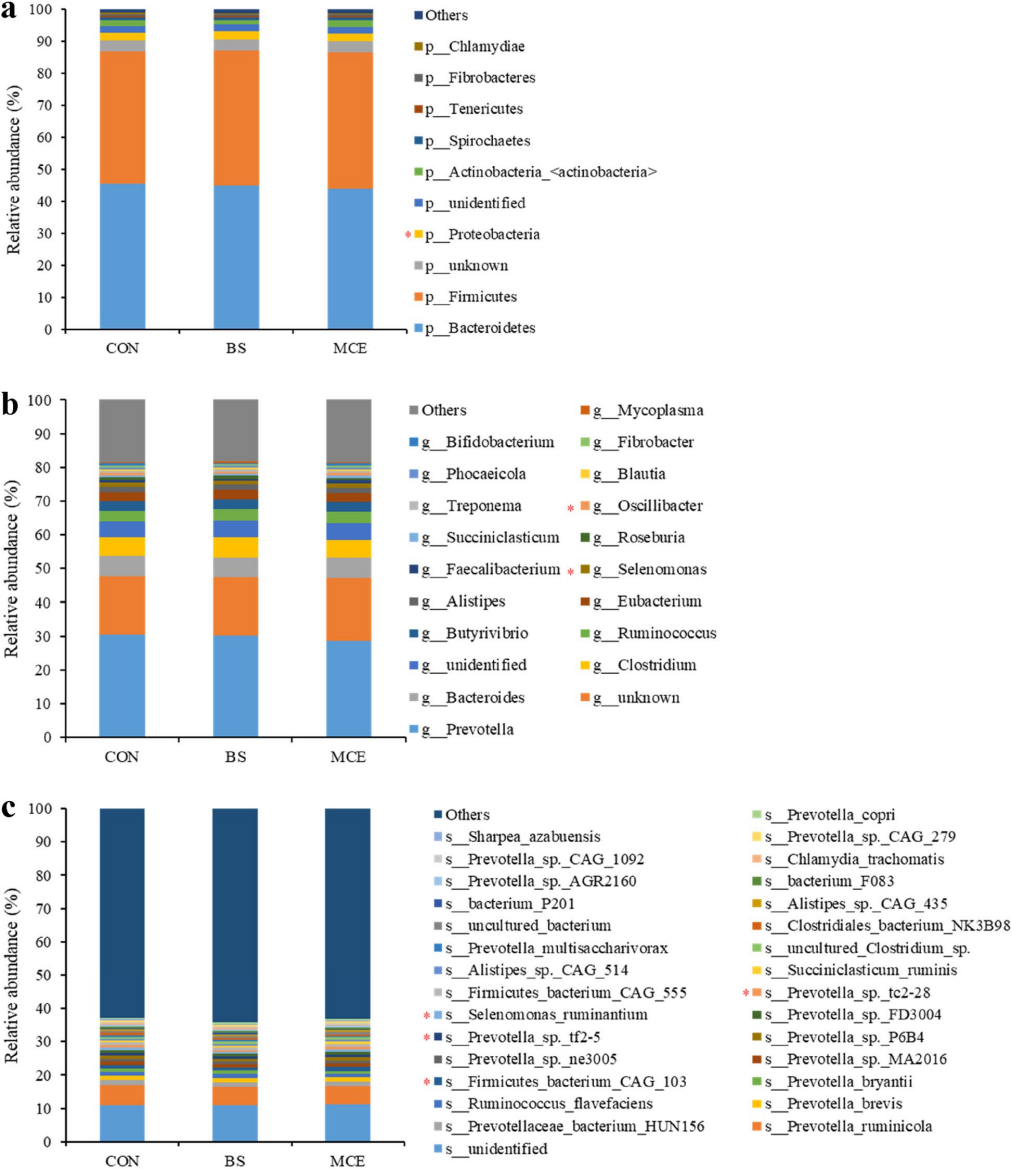
The relative abundances of bacteria in rumen. **a** Relative abundances of bacterial communities at the phylum level. **b** Relative abundances of bacterial communities at the genus level. **c** Relative abundances of bacterial communities at the species level. The difference among three groups was identified by Kruskal–Wallis multiple comparisons, and asterisk indicated the significant difference (*P* value < 0.05). CON control diet, BS control diet plus *Bacillus subtilis*, MCE control diet plus *Macleaya cordata* extract

The dominant bacterial phyla included Bacteroidetes (44.76 ± 4.90%), Firmicutes (41.96 ± 4.83%), and Proteobacteria (2.32 ± 0.62%); the dominant bacterial genus was *Prevotella* (29.74 ± 3.89%), followed by *Bacteroides* (5.98 ± 0.98%), *Clostridium* (5.48 ± 1.03%), and *Ruminococcus* (3.30 ± 0.65%); and the dominant bacterial species included *Prevotella ruminicola* (5.75 ± 1.23%), *Prevotellaceae bacterium HUN156* (1.36 ± 0.29%), *Prevotella brevis* (1.36 ± 0.18%), *Ruminococcus flavefaciens* (1.10 ± 0.37%), and *Prevotella bryantii* (1.00 ± 0.46%). For differential abundance comparison analysis at the phylum level, the abundance of Proteobacteria was higher (*P* < 0.05) in the rumen of BS than MCE (Fig. 2a; Table S4). At the genus level, the abundance of *Selenomonas* was significantly lower in the rumen of BS cows than CON and MCE cows (*P* < 0.01), while the abundance of *Lachnospira* was significantly lower in the rumen of the MCE cows than CON and BS cows (*P* < 0.05). The *Oscillibacter* was more abundant in the MCE cows than BS cows (*P* < 0.05) (Fig. 2b; Table S4). At the species level, 2 species, including *Prevotella histicola* and *Prevotella disiens* exhibited higher (*P* < 0.01) abundances, while other 4 species (*Selenomonas ruminantium*, *Prevotella sp. tc2-28*, *Selenomonas sp. AE3005*, and *Corynebacterium stationis*) showed lower (*P* < 0.01) abundances in the rumen of BS than CON and MCE. Furthermore, *Firmicutes bacterium CAG 103, Prevotella sp. tf2-5*, and *Lachnospiraceae bacterium AD3010* exhibited lower (*P* < 0.01), while *Prevotella corporis* exhibited higher (*P* < 0.05) abundances in the rumen of BS than MCE (Fig. 2c; Table S4).

**Fig. 3:**
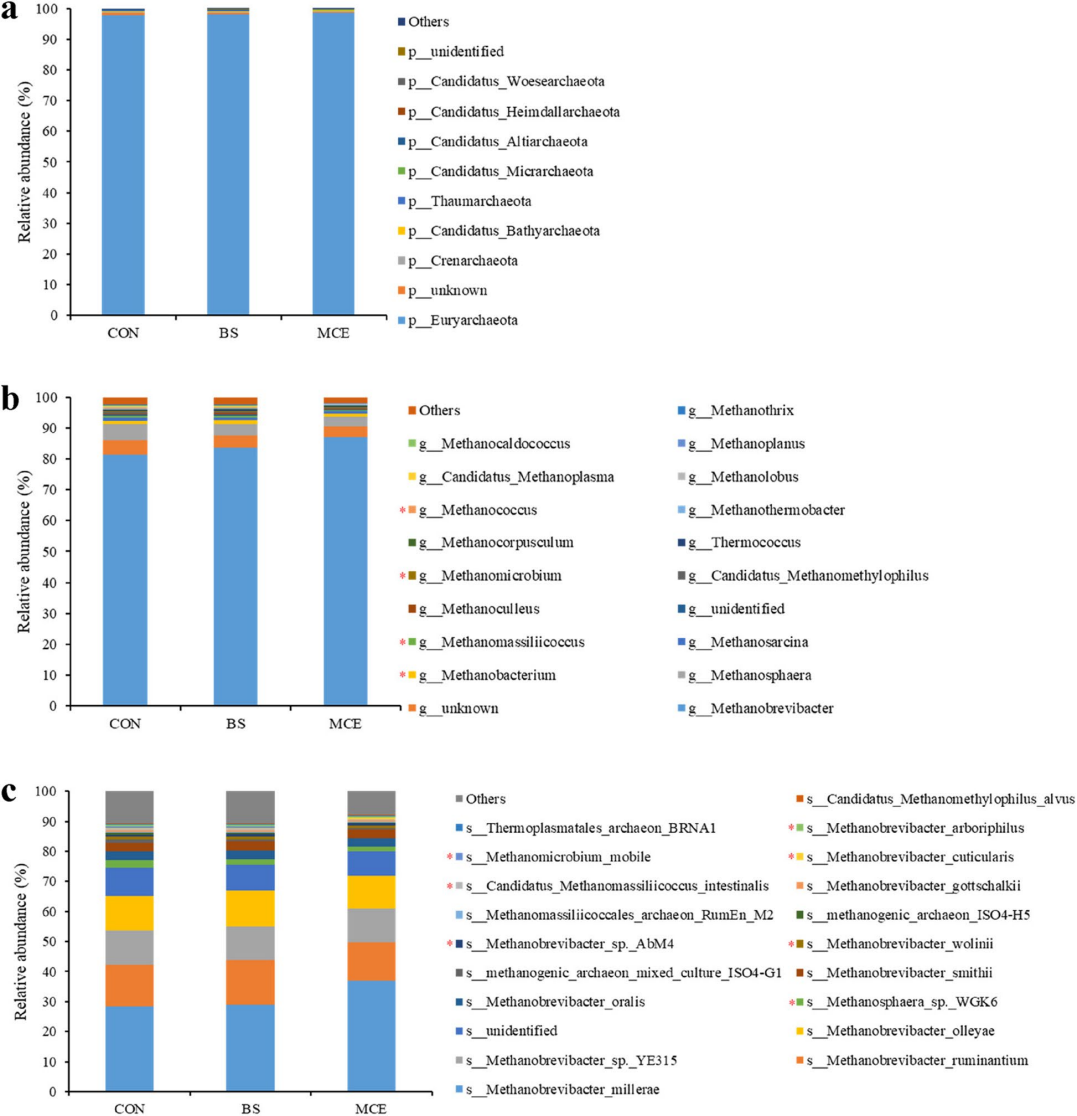
The relative abundances of archaea in rumen. **a** Relative abundances of archaeal communities at the phylum level. **b** Relative abundances of archaeal communities at the genus level. **c** Relative abundances of archaeal communities at the species level. The difference among three groups was identified by Kruskal–Wallis multiple comparisons, and asterisk indicated the significant difference (*P* value < 0.05). CON control diet, BS control diet plus *Bacillus subtilis*, MCE control diet plus *Macleaya cordata* extract

The most dominant archaeal phylum was Euryarchaeota (98.14 ± 0.87%); the dominant archaeal genera included *Methanobrevibacter* (84.09 ± 5.73%), *Methanosphaera* (4.16 ± 2.03%), and *Methanobacterium* (1.12 ± 0.27%); the dominant bacterial species included *Methanobrevibacter millerae* (31.45 ± 10.95%), *Methanobrevibacter ruminantium* (13.86 ± 3.04%), *Methanobrevibacter sp. YE315* (11.23 ± 2.93%), and *Methanobrevibacter olleyae* (11.46 ± 2.85%). For the differential abundance comparison analysis of archaea, no difference (*P* > 0.05) was found among groups at the phylum level (Fig. 3a; Table S4). At the genus level, six genera (*Methanobacterium*, *Methanomassiliicoccus*, *Methanomicrobium*, *Methanococcus*, *Methanoplanus*, and *Methanohalophilus*) had a lower abundance in the MCE cows than in the CON and BS cows (*P* < 0.05); *Methanosphaera* had a lower abundance in the MCE and BS cows than in the CON cows (*P* < 0.05) (Fig. 3b; Table S4). At the species level, seven species, including *Methanobrevibacter wolinii*, *Methanobrevibacter sp. AbM4*, *Candidatus Methanomassiliicoccus intestinalis*, *Methanobrevibacter cuticularis*, *Methanomicrobium mobile*, *Methanobacterium formicicum*, and *Methanobacterium congolense* showed lower (*P* < 0.01) abundances in the rumen of MCE than CON and BS; *Methanosphaera sp*. *WGK6* and *Methanosphaera stadtmanae* showed lower (*P* < 0.05) abundances in the rumen of MCE and BS than CON; four species (*Methanobrevibacter arboriphilus*, *Methanobrevibacter filiformis*, *Methanobrevibacter boviskoreani*, and *Methanobrevibacter curvatus*) exhibited lower (*P* < 0.05) abundances in the rumen of MCE than BS (Fig. 3c; Table S4).

Protozoa in the rumen produce most of the hydrogen in the rumen, which is the substrate for methanogenesis in the rumen. The protozoa are symbiotically associated with methanogens, so the protozoa indirectly affect rumen methanogenesis. However, there were no significant differences (*P* > 0.05) in the abundance of the ciliate protozoa among the three groups (Table S5).

### Functional profiles of the rumen microbiome and differential functions among cows in three groups

#### Functional profiles of the rumen microbiome

**Fig. 4:**
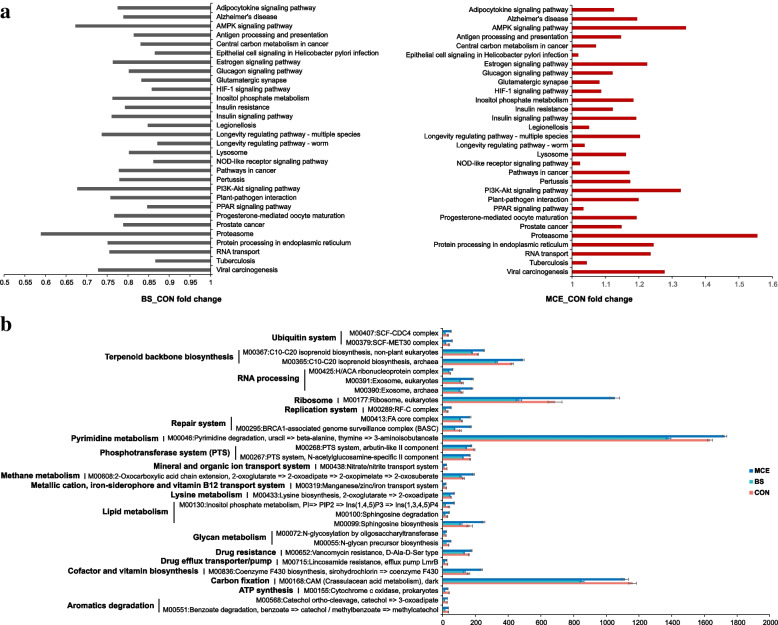
Differential KEGG functions among CON, BS, and MCE cows. **a** Cows fold change of significantly enriched metabolic pathways. **b** Comparison of rumen microbial KEGG modules among CON, BS, and MCE cows. The Kruskal–Wallis multiple comparisons was used for mean comparison. CON control diet, BS control diet plus *Bacillus subtilis*, MCE control diet plus *Macleaya cordata* extract

The Kyoto Encyclopedia of Genes and Genomes (KEGG) profiles and the genes encoding CAZymes were used to identify the functions of the cows’ rumen microbiome. The metagenomic sequences were mapped to 165 KEGG third-level pathways which were considered as rumen microbial metabolic pathways. These pathways belonged to six first-level categories, including “Metabolism” (63.04 ± 4.78%), “Genetic Information Processing” (14.46 ± 0.83%), “Environmental Information Processing” (6.41 ± 1.01%), “Organismal Systems” (5.82 ± 2.30%), “Human Diseases” (5.65 ± 1.47%), and “Cellular Processes” (4.61 ± 0.64%). At the second level, 46 categories were observed, with “Carbohydrate metabolism” (16.07 ± 1.12%), “Global and overview maps” (10.39 ± 0.85%), “Amino acid metabolism” (8.58 ± 0.73%), “Nucleotide metabolism” (7.54 ± 0.57%), “Replication and repair” (6.80 ± 0.68%), and “Energy_metabolism” (6.33 ± 0.49%) being the most abundant. When comparing the KEGG pathways identified in the BS and CON groups, a total of top 30 significantly different level-3 pathways, including 10 “Human Diseases” pathways, 10 “Organismal Systems”, six “Cellular Processes”, three “Environmental Information Processing”, and one “Genetic Information Processing” pathways, were enriched in the rumen microbiomes of the CON cows (*P* < 0.05; Fig. 4a). However, when comparing MCE and CON groups, these pathways were enriched (*P* < 0.05) in the MCE group (Fig. 4a). Protein, energy, and fat are indispensable sources of nutrition for dairy cows. In addition, CH_4_ emissions are closely related to energy utilization. We further selected the key pathways involved in amino acid metabolism, energy metabolism, carbohydrate metabolism, and lipid metabolism. Only one pathway, “Inositol phosphate metabolism” was more abundant (*P* < 0.05) in the rumen of the MCE cows than BS cows (Fig. S1). When the top 30 significantly different (*P* < 0.05) KEGG modules were compared, 20 modules were enriched in the rumen microbiomes of the MCE cows compared with BS cows, five modules were enriched in the rumen microbiomes of the CON and MCE cows compared with BS cows, three modules were enriched in the rumen microbiomes of the CON cows compared with BS cows, and two modules were enriched in the rumen microbiomes of the MCE cows compared with CON and BS cows (Fig. 4b).

**Fig. 5:**
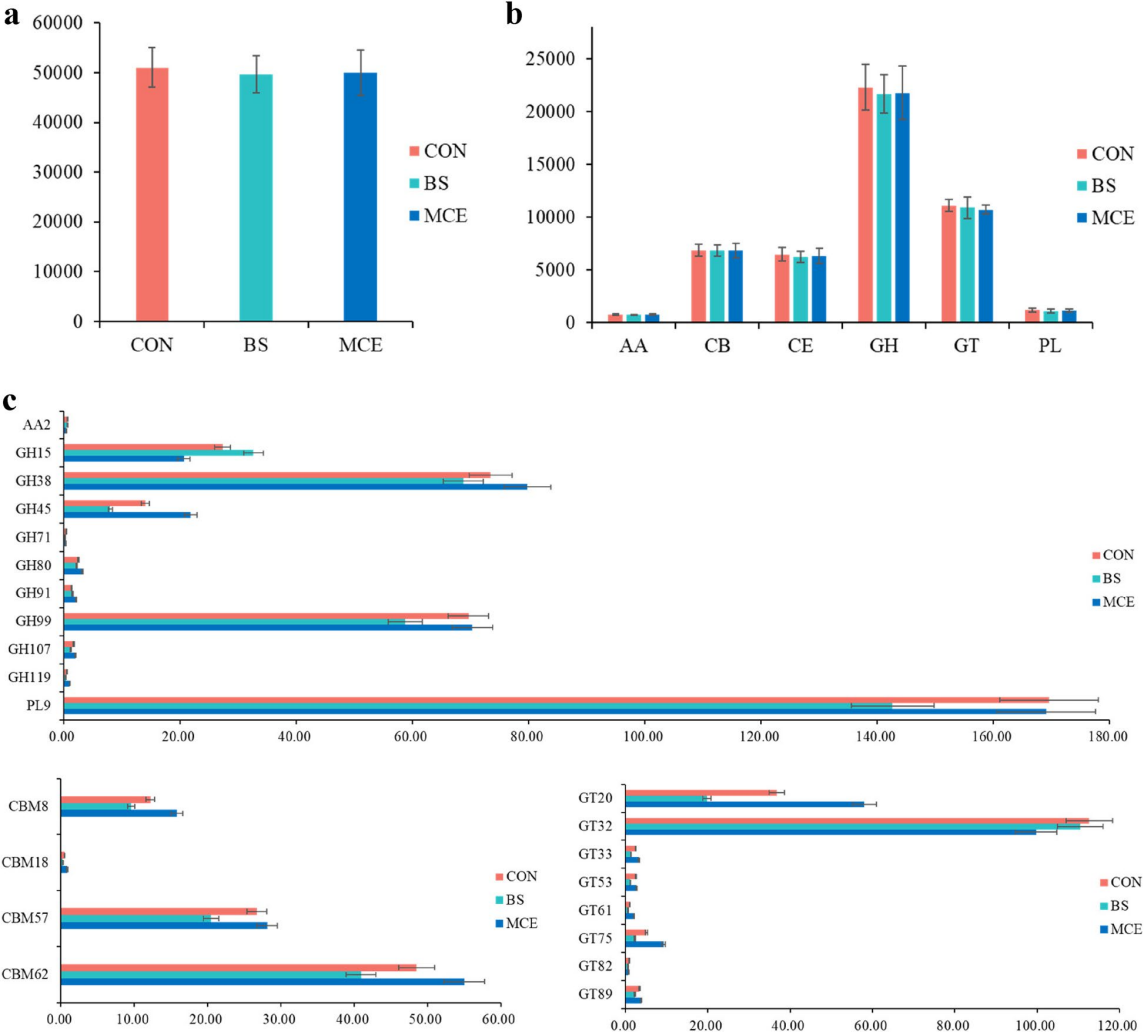
**a** Comparisons of the total abundance of CAZymes genes of rumen microbiomes of cows. **b** Comparisons of the abundance of the CAZymes gene families of the rumen microbiomes of cows. **c** Comparisons of the gene abundance of the CAZymes families (GH, CE, PL, and AA; CBM; GT) of the rumen microbiomes of cows; only families with significant differences among groups were shown. The Kruskal–Wallis multiple comparisons was used for mean comparison, and the *P* value < 0.05 indicated the significant difference. CON control diet, BS control diet plus *Bacillus subtilis*, MCE control diet plus *Macleaya cordata* extract

For CAZyme profiles, a total of 310 genes encoding CAZymes were searched. including seven auxiliary activities (AAs), 65 carbohydrate-binding modules (CBMs), 16 carbohydrate esterases (CEs), 115 glycoside hydrolases (GHs), 87 glycosyltransferase (GTs), and 20 polysaccharide lyases (PLs). The BS or MCE showed no difference (*P* > 0.05) in the abundance of CAZymes with respect to the CON (Fig. 5a). Among those six classes of CAZymes families (AA, CBM, CE, GH, GT, and PL), there were also no differences (*P* > 0.05) in the abundance of genes belonging to those six classes (Fig. 5b). Among the genes encoding CAZymes involved in deconstructing carbohydrates (including cellulose, hemicellulose, starch, protein, and lignin), one was enriched (*P* < 0.05) in the rumen of MCE than CON, one was enriched (*P* < 0.05) in the rumen of CON and BS than MCE, two were enriched (*P* < 0.05) in the rumen of CON and MCE than BS; two were enriched (*P* < 0.05) in the rumen of BS than MCE, while five were enriched (*P* < 0.05) in the rumen of MCE than BS (Fig. 5c; Table S6). Among the CBMs, the noncatalytic CAZymes that are involved in the degradation of complex carbohydrates, four were enriched (*P* < 0.05) in the rumen of MCE than BS (Fig. 5c; Table S6). Regarding the GTs (involved in carbohydrate synthesis), one was enriched (*P* < 0.05) in the rumen of CON than BS, one was enriched (*P* < 0.05) in the rumen of CON than MCE, two were enriched (*P* < 0.05) in the rumen of CON and MCE than BS, and four were enriched (*P* < 0.05) in the rumen of MCE than BS (Fig. 5c; Table S6).

#### Carbohydrate genes related to fiber and starch degradation pathway

**Fig. 6:**
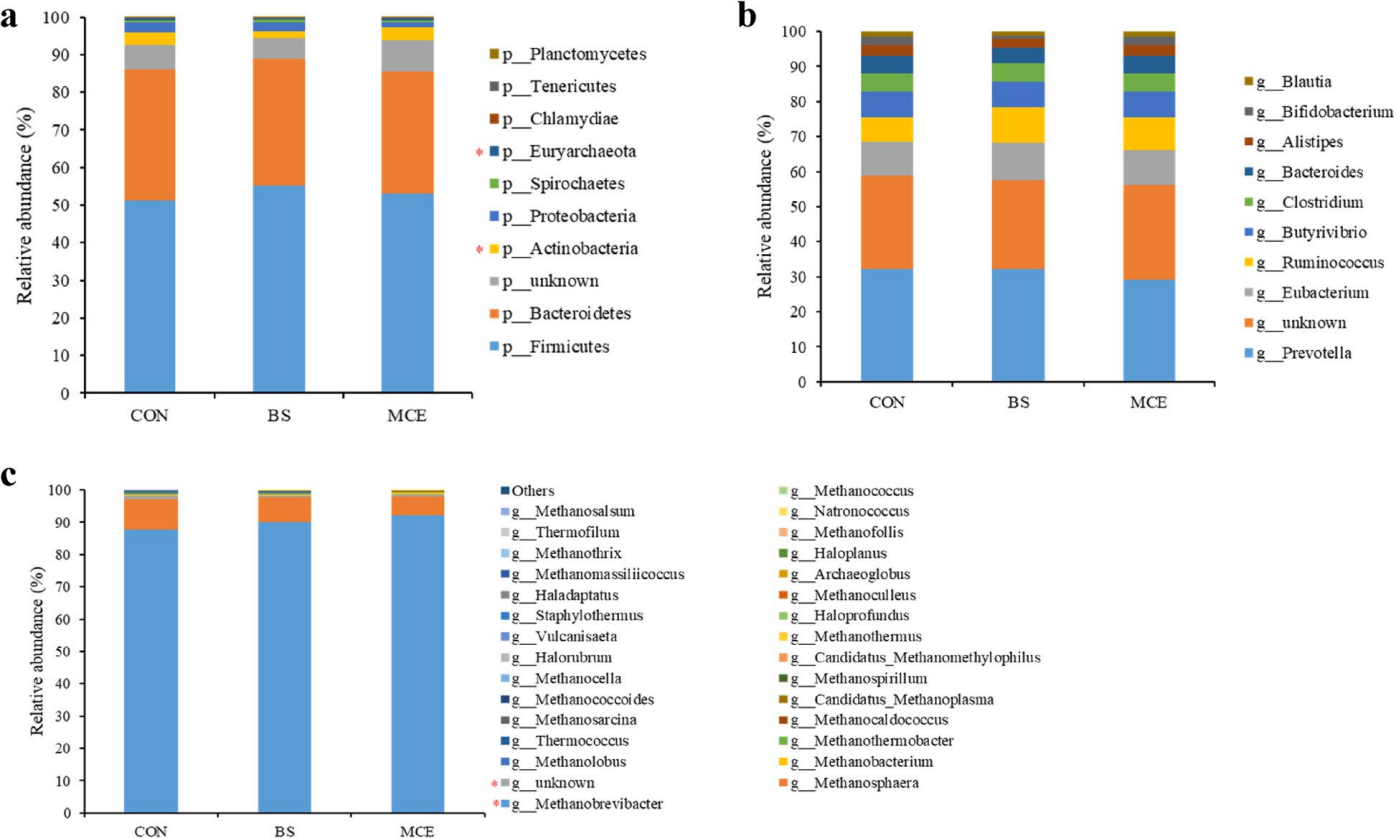
Phylogenetic distribution of sequences in GH13 assigned to the identified phylum (**a**) and genus (**b**). **c** Relative abundances of archaeal communities involved in methane metabolism at the genus level. The Kruskal–Wallis multiple comparisons was used for mean comparison, and asterisk indicated the significant difference (*P < 0.05). CON control diet, BS control diet plus *Bacillus subtilis*, MCE control diet plus *Macleaya cordata* extract

In order to provide support for further explore the effects of BS and MCE on pivotal fiber biodegradation processes, we screened for fibrinolytic enzymes, including cellulase, hemicellulase, and ligninase. We found these three enzymes were grouped into the 65 families of GHs. In particular, among the gene encoding enzymes, the relative abundances of the GH38 and GH45 families were higher in the MCE group than the BS group (*P* < 0.05), while had no differences (*P* > 0.05) between these two additives groups and the CON group (Table S7). Comtet-Marre et al. [29] demonstrated that GH13 is a representative family of CAZymes in the rumen of dairy cows. Moreover, in the GH families associated with amylolytic enzymes, the starter feed only increased the abundance of genes coding GH13 in lambs [30]. In order to explore the effects of BS and MCE on pivotal starch biodegradation process, we investigate GH13 in more detail. According to the non-redundant genes annotated to this enzyme family, these non-redundant genes were then annotated to obtain the corresponding bacterial species and abundance information. The GH13 were mainly assigned to Firmicutes at the phylum level; *Prevotella* and *Eubacterium* were the most assigned genera (Fig. 6a,b). Furthermore, the phylum Euryarchaeota showed higher (*P* < 0.05) abundance in the rumen of CON and MCE than BS cows, and Actinobacteria showed higher (*P* < 0.05) abundance in the rumen of MCE than BS cows (Fig. 6a,b).

#### The fermentation pathways from glucose into acetate, propionate, and butyrate by microorganisms

After fiber and starch degradation pathways, many gene encoding enzymes were involved in VFA fermentation pathways of microorganisms. The production of acetate and butyrate releases H_2_, which can become an energy source of methanogens. However, the production of propionate can compete with methanogens for H_2_ and thus reduce enteric CH_4_ emissions. We screened for the fermentation pathway of metabolizing glucose into acetate, propionate, and butyrate, which involved 32 encoding enzymes. There were no significant differences (*P* > 0.05) in the abundance of the 32 enzymes were observed between the two additive groups and the CON group (Fig. S2).

#### Potential microbial interactions identified in co‑occurrence networks

**Fig. 7:**
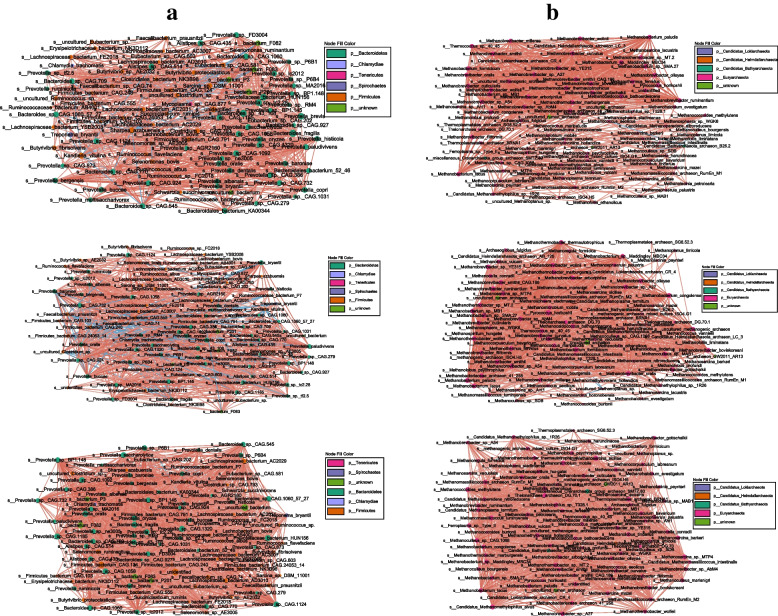
**a** The co‑occurrence among rumen bacteria of cows in the CON, BS, and MCE groups. **b** The co‑occurrence among rumen archaea of cows in the CON, BS, and MCE groups. Only significant (*P* < 0.05) relationships are shown. Red edges indicate positive relationships, and blue edges indicate negative relationships. The node size is proportional to the mean abundance. CON control diet, BS control diet plus *Bacillus subtilis*, MCE control diet plus *Macleaya cordata* extract

Co-occurrence network among rumen bacteria analysis revealed a total of 3340 co-occurrence relationships, with distinct co-occurrence patterns being found in each group of the cows (Fig. 7a**)**. In the rumen bacteria of the CON cows, 881 connections (*P* < 0.05) were found, with the most positive relationships existing among taxa of Bacteroidetes and the most negative relationships existing between taxa of Firmicutes and taxa of Bacteroidetes. The 952 connections (*P* < 0.05) were found in the rumen bacteria of BS cows, among which the taxa of Bacteroidetes had the most positive relationships, while the taxa of Bacteroidetes and taxa of Firmicutes had the most negative relationships. In the rumen bacteria of the MCE cows, 1507 connections (*P* < 0.05) were found, with taxa of Bacteroidetes positively correlated with each other but negatively correlated with *Ruminococcaceae bacterium P7*.

Co-occurrence network among rumen archaea analysis revealed a total of 3512 co-occurrence relationships (Fig. 7b). In the rumen archaea of the CON cows, 1000 connections (*P* < 0.05) were found, with the most positive relationships existing among taxa of Euryarchaeota, the *Candidatus Bathyarchaeota archaeon B26.2* negatively correlated with *Methanococcus aeolicus*, *Methanosphaera sp*. *WGK6*, and *Methanoculleus sp*. *SDB*. In the rumen archaea of BS cows, 1255 connections (*P* < 0.05) were found, with the most positive relationships existing among taxa of Euryarchaeota. The *uncultured Methanoplanus sp*. negatively correlated with *Candidatus Methanomassiliicoccus intestinalis*, *Methanohalophilus sp*. *T328.1*, and *Methanosarcina horonobensis*. The *methanogenic archaeon ISO4.H5* negatively correlated with *Methanolobus psychrophilus*. In the rumen archaea of the MCE cows, 1257 connections (*P* < 0.05) were found, and all taxa were positively correlated.

**Fig. 8:**
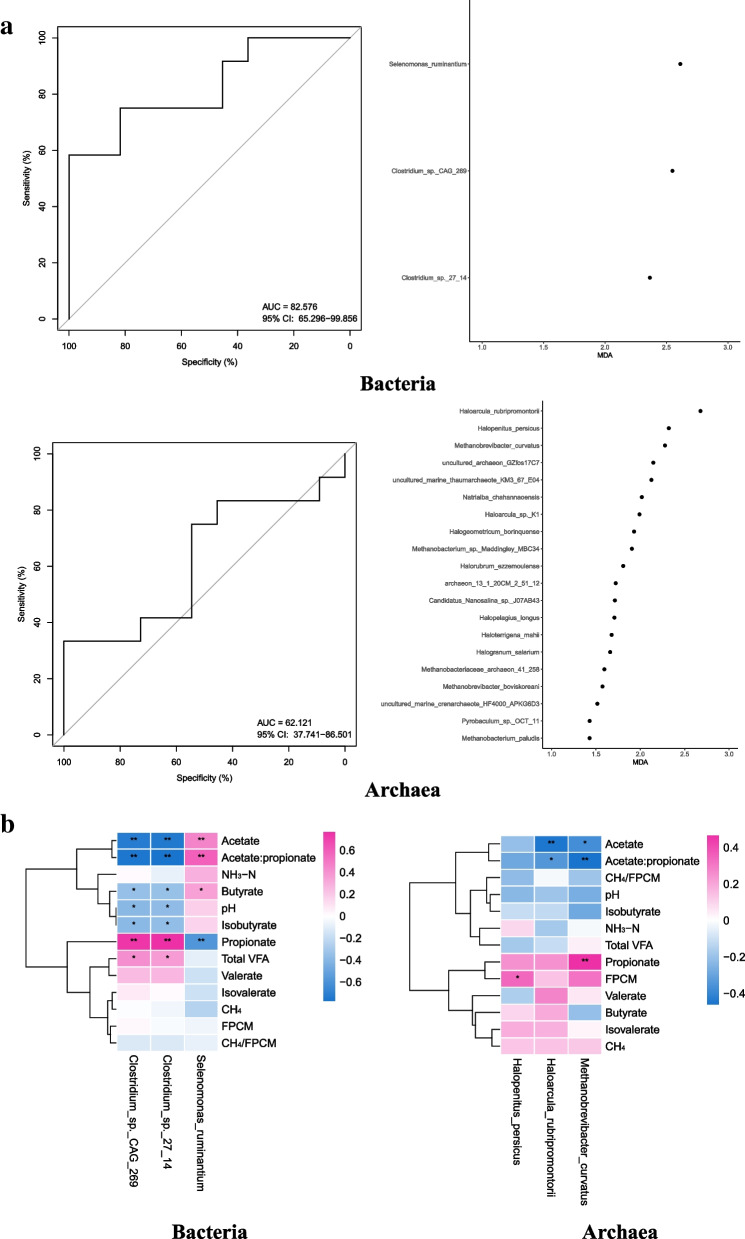
**a** Receiver operating characteristic (ROC) curve and the confusion matrix of the performance of the random forest model using the top 20 microbiomes based on mean decrease accuracy (only three species of bacteria were obtained). **b** Heatmaps display the Spearman’s correlation coefficients among rumen microbiomes, cow phenotypes, and rumen fermentation characteristics. *P < 0.05, **P < 0.01. CON control diet, BS control diet plus *Bacillus subtilis*, MCE control diet plus *Macleaya cordata* extract

The random forest model and heatmaps were performed to identify the rumen microbiomes that were related to cow phenotypes and rumen fermentation characteristics (Fig. 8a,b). Of the identified bacterial biomarkers by the random forest model and heatmaps, both *Clostridium sp*. *CAG* 269 and *Clostridium sp*. *27 14* were negatively associated (*P* < 0.05) with acetate, butyrate, acetate:propionate ratio, isobutyrate, and pH value, while *Selenomonas ruminantium* was positively associated (*P* < 0.05) with acetate, butyrate, and acetate:propionate ratio. Furthermore, both *Clostridium sp*. *CAG* 269 and *Clostridium sp*. *27 14* were positively associated (*P* < 0.05) with propionate and total VFA, while *Selenomonas ruminantium* was negatively associated (*P* < 0.05) with propionate. Of the identified archaeal biomarkers by the random forest model and heatmaps, both *Haloarcula rubripromontorii* and *Methanobrevibacter curvatus* were negatively associated (*P* < 0.05) with acetate, butyrate, and acetate:propionate ratio. However, *Halopenitus persicus* was positively associated (*P* < 0.05) with FPCM, and *Methanobrevibacter curvatus* was positively associated (*P* < 0.05) with propionate.

## Discussion

Our results indicated that BS and MCE increased FPCM yields, while decreased CH_4_ emissions per kilogram of FPCM. Both additives have been shown to benefit the health of cattle, BS improved digestion and increased serum IgG and IFN-γ levels [31, 32] and MCE improved intestinal morphology and reduced the incidence of respiratory diseases based on its antibacterial and anti-inflammatory effects [33, 34]. In agreement with our results, Choonkham et al. and Souza et al. [35, 36] also reported that BS increased milk yield in dairy cows. To our knowledge, there were few studies on MCE in dairy cows. Previous researches have shown that MCE reduces small intestinal damage and respiratory morbidity in young animals [34, 37]. Therefore, it could be speculated that the observed improvements in lactation performance may be due to the anti-inflammatory and immune boosting capability of MCE. In the present study, BS lowered the acetate proportion, but increased the propionate proportion. Similar results have been observed in previous studies with dairy cows and calves [9, 38]. VFA can provide ruminants with more than 70% of their digestive energy [39], and a higher concentration of propionate can increase milk production [40]. The alteration in propionate in this study may indirectly explain the increased FPCM yield in the BS group. BS affected the molar proportion of VFA components in the rumen of dairy cows, such as reducing the acetate:propionate ratio, which could explain the reduction of CH_4_ emissions in the BS group.

The CH_4_ emissions and lactation performance of dairy cows are largely affected or determined by the rumen microbiome [41, 42]. In the present study, we deeply dissected the effects of BS and MCE on CH_4_ emissions, ruminal microbial composition, and functions, contributing to clarifying the effect of additives on methanogenesis in a lactating dairy cow model.

*Selenomonas* are regarded as starch-degrading bacteria, and most of their strains can utilize starch to produce acetate, succinate, and propionate [43, 44]. A previous study demonstrated that higher molar proportion of propionate in cattle than in yaks, as well as the level of *Selenomonas* [45]. Inconsistent with this finding, in the present study, the abundance of *Selenomonas* in the BS was lower than that in the CON, but the molar proportion of propionate was higher. The species *Selenomonas ruminantium* and *Selenomonas sp. AE3005* belonging to the genus of *Selenomonas* also exhibited lower abundances in the BS than CON. The results of the random forest model and heatmaps showed that the abundance of *Selenomonas ruminantium* was negatively correlated with the molar proportion of propionate, while positively correlated with the molar proportions of acetate and butyrate, and the acetate: propionate ratio. Ramayo-Caldas et al. [46] demonstrate that cows with greater CH_4_ yield had a higher abundance of *Lachnospiraceae* in the rumen. In the current study, the genus *Lachnospira* was more abundant in the CON cows than in the MCE cows, which could explain, at least in part, the lower CH_4_ intensity in the MCE cows than in the CON cows.

It has been previously reported that *Metanobrevibacter* is the predominant methanogen in the rumen, followed by *Metanosphaera*, *Methanobacterium*, and *Metanomassilicoccals* [47, 48]. *Methanosphaera* is an obligate H_2_-dependent methanol-utilizing methanogen, *Metanomassilicoccals* and *Methanohalophilus* are methanol- and methylamine-reducing methanogens. The genera *Metanobrevibacter*, *Methanobacterium*, *Methanomicrobium*, *Methanococcus*, and *Methanoplanus* belong to hydrogenotrophic methanogens, which form CH_4_ through the reduction of CO_2_. *Metanobrevibacter* was also the predominant methanogen in the present study. However, Söllinger et al. [49] reported *Methanosphaera* and *Metanomassilicoccals* may account for a larger share of overall CH_4_ production than previously thought, compared to CO_2_-reducing *Metanobrevibacter*. It was also shown that CH_4_ emissions in cows are positively correlated with *Methanosphaera* and *Metanomassilicoccals*, though not with CO_2_-reducing methanogens (*Metanobrevibacter*) [49]. The genera *Metanosphaera*, *Methanobacterium*, *Metanomassilicoccals*, *Methanomicrobium*, *Methanococcus*, *Methanoplanus*, and *Methanohalophilus* had a lower abundance in the MCE cows than in the CON cows, which could support that MCE decreased CH_4_ intensity. The lower abundances of *Metanosphaera*, *Methanosphaera sp*. *WGK6*, and *Methanosphaera stadtmanae* in the BS group also support the reduction of methane intensity by BS. We obtained the non-redundant genes involved in CH_4_ metabolism and annotated these non-redundant genes to obtain information about the corresponding archaeal genera and their abundance (Fig. 6c). These indicated the highest relative abundance of *Methanobrevibacter*, and the abundance in the MCE cows was higher than that in the CON cows, which may be due to the reduction of the other seven archaeal genera in the MCE cows.

Metagenomics identified no differences in amino acid metabolism, energy metabolism, carbohydrate metabolism, and lipid metabolism pathways in the present study. Similarly, Xue et al. [50] reported that metagenomics did not detect differences in the abundance of the top 20 active pathways between low- and high-feed-efficiency dairy cows, whereas metatranscriptomics detected differences in 10 of these pathways. Focusing on the pathway of methane metabolism, no differences in the abundance of modules enzymes were found. This is inconsistent with the results of the relative abundance comparison analysis of archaea, which found that MCE reduced the relative abundance of seven archaeal genera and nine archaeal species. Taxis et al. [51] showed that microorganisms of different taxonomic level in the rumen could share the similar metabolic functions. Previous studies in humans first reported that the functional profiles of the microorganisms were more conserved than the taxonomic composition [52, 53]. Li et al. [54] indicated that the degrees of association between feed efficiency and rumen microbial functional characteristics or taxonomic characteristics were different. The above findings may suggest the inconsistent results of the abundance of enzymes and archaea. Inconsistent with our findings, Roehe et al. [55] observed that CH_4_ emissions were less associated with rumen microbial taxonomic profiles than functional profiles. Therefore, analysis of rumen microbial community or functional features alone may be insufficient to find a true biological linkage between the rumen microbiome and methanogenesis.

Compared with BS cows, genes encoding CAZymes involved in deconstructing carbohydrates (GH77, GH99) in the rumen microbiomes were enriched in the CON cows. This result did not indicate that BS improved the ability of cows to degrade complex substrates. Although both BS and MCE decreased the gene abundance of the CAZymes involved in carbohydrate synthesis (BS: GT53, GT82, GT89; MCE: GT32) compared with the CON group, neither additive affected ruminal total VFA concentration in dairy cows. BS and MCE did not affect the gene abundance of the GH family gene-coded fibrinolytic enzymes in the current study. Firmicutes plays an important role in the degradation of carbohydrates, including starch, cellulose, hemicellulose, and oligosaccharides [56]. *Prevotella* is involved in the degradation of starch, hemicellulose, and pectin [57]. The previous study demonstrated that the abundance of *Prevotella* was the highest in CAZymes in the rumen of dairy cows [58]. Guo et al. and Park et al. [59, 60] reported that Firmicutes was enriched in dairy cows fed high energy diets rich in starch. Similarly, the GH13, associated with the starch biodegradation process, was mainly assigned to the phylum of Firmicutes, and the genera of *Prevotella* and *Eubacterium* in the present study. Liu et al. [61] showed that Actinobacteria was also a primary bacteria involved in the degradation of starch; however, BS reduced the role of Actinobacteria in the starch biodegradation process. There were no significant differences in the abundance of a total of 32 enzymes discovered at the metagenomic level between the additive groups and CON group when the fermentation process from glucose to acetate, propionate, and butyrate was studied. The decrease in acetate and butyrate proportions, and the increase in propionate proportion in the BS group, were not attributed to the shift in the catalysis of various enzymes involved in the fermentation pathway. These findings suggest that BS altered the composition and function of the rumen microbiomes, thereby stabilizing the microbial ecosystem, and producing less acetate and butyrate, and more propionate.

As we all know, the phyla Bacteroidetes and Firmicutes are the dominant bacteria in the rumen of dairy cows [62]. This indicates that Bacteroides and Firmicutes play a key role in feed digestion and nutrient metabolism of dairy cows. Some previous studies have shown that the Bacteroidetes is more abundant in the rumen of dairy cows fed high-forage or low-energy diets, than the Firmicutes, which is more abundant in the rumen of dairy cows fed high-energy diets [58, 60]. Generally, Bacteroidetes is a net H_2_ utilizer, whereas Firmicutes is an H_2_ producer [63, 64]. In the co-occurrence network analysis, the differences found above between the Bacteroidetes and Firmicutes supported the negative correlation between these two bacterial phyla. The previous study indicated that predominant cellulose degraders were mainly assigned with Bacteroidetes in the non-methane-excreting individuals; however, Firmicutes was the most assigned bacteria in the methane-excreting subjects [65]. These previous studies showed that the enrichment of Firmicutes appeared to represent more CH_4_ production. However, at a high concentrate level, the relative abundance of the Firmicutes was increased in the rumen, whereas that of Bacteroidetes was increased in the rumen at a high forage level [56, 58]. In addition, the Firmicutes-to-Bacteroidetes ratio increased, indicating improved energy acquisition in ruminants [56]. Therefore, the Firmicutes-to-Bacteroidetes ratio was regarded as an index to reflect the utilization of feed in ruminants [66]. Further, Jami et al. [67] reported that the Firmicutes-to-Bacteroidetes ratio was positively correlated with feed efficiency, milk yield, and milk fat yield in dairy cows. Consistent with these findings, both BS and MCE increased the Firmicutes-to-Bacteroidetes ratio compared with CON (0.94 vs 0.97 vs 0.91), which suggested the increase of FPCM production in BS and MCE cows.

*Faecalibacterium prausnitzii* had the highest number of negative correlations among bacteria of BS cows, and these negative correlations were associated with species belonging to the genus of *Prevotella*. This result could be attributed to *Faecalibacterium prausnitzii* is a butyrate-producing bacteria [68], while *Prevotella* is an acetate- and propionate-producing bacterium [69]. Notably, the negative correlations among bacteria were more obvious in the MCE group, concentrated in the *Ruminococcaceae bacterium P7* belongs to the phylum of Firmicutes and the *Prevotella* belongs to the phylum of Bacteroidetes. *Ruminococcus* and *Prevotella* are both major contributors to CAZymes and were found in the GH, GT, CBM, CE, and PL families [58]. The previous studies reported that *Ruminococcus* was highly negatively correlated with feed conversion efficiency, while *Prevotella* was enriched in dairy cows with high feed conversion efficiency [70, 71]. It has been shown that *Ruminococcus* may be an important contributor to hemicellulose degradation, but appears to have little effect on oligosaccharide degradation [58]. Zened et al. [72] indicated that *Prevotella* does not degrade cellulose, however, is a contributor to the degradation and utilization of starch and plant cell wall polysaccharides such as xylan and pectin. Furthermore, *Ruminococcus* was more abundant in the rumen of dairy cows fed high-forage diets, whereas *Prevotella* was more abundant under low-forage diets [58]. *Ruminococcus* and *Prevotella* were associated with enteric CH_4_ emissions; specifically, *Ruminococcus* was enriched in sheep with high CH_4_ yield, whereas *Prevotella* was negatively correlated with CH_4_ emissions [73, 74]. The negative correlation between the *Ruminococcus* and *Prevotella* in the present study can be explained as shown above, but the role at the species level is unclear and needs to be interpreted with caution. In the present study, the co-occurrence network among rumen archaea analysis revealed that almost all correlations exist among taxa of the Euryarchaeota, and almost no negative correlations existed. The archaeal communities are dominated by the phylum Euryarchaeota, and the average relative abundance of Euryarchaeota was 98.1% in the present study. Moissl-Eichinger et al. [75] reported that, essentially, Euryarchaeota interact based on three driving drivers, specifically the environmental pressure, the capability for the exchange of metabolites/electrons, and the adaptability of the genome and structure. Both BS and MCE showed more connections in the bacterial or archaeal co-occurrence network than the CON cows, which suggested these additives increase microbe-microbe interactions in the microbiomes in the present study.

*Selenomonas ruminantium* is an amylolytic bacteria in the rumen, which is involved in the conversion of succinate to propionate [76]. Inconsistent with this finding, *Selenomonas ruminantium* was negatively correlated with propionate but positively correlated with acetate and butyrate in the present study. Moreover, the random forest model and heatmaps revealed that the species *Clostridium*_*sp.*_*CAG*_*269* and *Clostridium*_*sp.*_*27_14* belong to the genus of *Clostridium* were positively correlated with total VFA and propionate, however, negatively correlated with acetate, butyrate, acetate:propionate ratio, isobutyrate, and pH value in the present study. Not similar to our findings, some previous studies showed that *Clostridium* can produce butyrate, which is a cellulolytic bacterium and is positively correlated with the acetate:propionate ratio [77–79]. However, there is little specific information about these two bacterial species. In the archaea, we found that *Methanobrevibacter curvatus*, *Haloarcula rubripromontorii*, and *Halopenitus persicus* were related to the proportion of VFA, as well as FPCM yield. *Methanobrevibacter curvatus* was originally isolated from termites and positively correlated with CH_4_ production [80, 81]. Sánchez et al. [82] drafted the genome of *Haloarcula rubripromontorii*; *Halopenitus persicus* was isolated from the salty lake [83]. Beyond the above information, there is little literature describing these three archaeal species. Although the use of microorganisms at the specie level for random forest analysis allowed us to obtain less information about these microorganisms, it improved the accuracy, and these mechanisms will gradually be clarified with further research in the future.

## Conclusions

The present result shows that both BS and MCE additives improved FPCM yield, while reduced CH_4_ intensity (CH_4_/FPCM, g/kg) in dairy cows. BS decreased molar proportions of acetate and butyrate but increased the molar proportion of propionate. MCE reduced relative abundances of seven archaeal genera and nine archaeal species, and BS reduced relative abundances of one archaeal genus and two archaeal species. These results suggest that the mitigation function of BS and MCE on ruminal methanogenesis might be attributed mainly to their effects on VFA production and the relative abundance of archaea in the rumen. The co‑occurrence network analysis of rumen bacteria revealed that interaction patterns were different among dietary treatments, and the MCE cows had the most microbe–microbe associations in bacteria. On the other hand, the co‑occurrence network analysis of rumen archaea found that there was almost no negative correlation among taxa, and both BS and MCE cows had more microbe–microbe associations in archaea than the CON cows. The random forest and heatmaps analysis revealed correlations between the six microbial species with cow phenotypes and rumen fermentation characteristics.

### Supplementary Information

**Additional file 1: Table S1.** Ingredient and chemical composition of the basal diet fed to dairy cows. **Table S2.** Summary of sequence data generated from rumen samples. **Table S3.** Comparison of microbial domains among CON, BS or MCE cows. **Table S4.** The relative abundances of differential rumen bacteria and archaea among CON, BS or MCE cows. **Table S5.** The relative abundances of protozoa in rumen of dairy cows. **Table S6.** The CAZymes families with significant difference in gene abundance among three groups. **Table S7.** The relative abundances of GH family genes coded fibrolytic enzymes. **Figure S1.** Fold changes of metabolic pathways identified in the metagenomes of the cows. **Figure S2.** Comparisons of the abundance of KO enzymes related to the acetate, propionate, and butyrate production pathway of cows. The Kruskal–Wallis multiple comparisons was used for mean comparison, and asterisk indicated the significant difference (*P* < 0.05). CON = control diet; BS = control diet plus *Bacillus subtilis*; MCE = control diet plus *Macleaya cordata* extract.

## Data Availability

The rumen metagenome sequences were deposited into NCBI Sequence Read Archive (SRA) under the accession number of PRJNA981443.
