# Supplementary material for: Bacillus subtilis and Macleaya cordata extract regulate the rumen microbiota associated with enteric methane emission in dairy cows
Source: Microbiome. 2023 Oct 19;11:229. doi: 10.1186/s40168-023-01654-3 (PMC10585854; doi:10.1186/s40168-023-01654-3)
Supplement: Supplementary file 2 — Additional file 1: Table S1. Ingredient and chemical composition of the basal diet fed to dairy cows. Table S2. Summary of sequence data generated from rumen samples. Table S3. Comparison of microbial domains among CON, BS or MCE cows. Table S4. The relative abundances of differential rumen bacteria and archaea among CON, BS or MCE cows. Table S5. The relative abundances of protozoa in rumen of dairy cows. Table S6. The CAZymes families with significant difference in gene abundance among three groups. Table S7. The relative abundances of GH family genes coded fibrolytic enzymes. Figure S1. Fold changes of metabolic pathways identified in the metagenomes of the cows. Figure S2. Comparisons of the abundance of KO enzymes related to the acetate, propionate, and butyrate production pathway of cows. The Kruskal–Wallis multiple comparisons was used for mean comparison, and asterisk indicated the significant difference (P < 0.05). CON = control diet; BS = control diet plus Bacillus subtilis; MCE = control diet plus Macleaya cordata extract. [file 40168_2023_1654_MOESM1_ESM.docx]

**Table S1** Ingredient and chemical composition of the basal diet fed to dairy cows

| Item | Value, g/kg DM |
| --- | --- |
| Ingredient composition | |
| Corn silage | 247 |
| Alfalfa hay | 153 |
| Oatgrass hay | 30 |
| Steam-flaked corn | 120 |
| Soybean meal | 114 |
| Corn flour | 140 |
| Beet pulp | 44 |
| Whole cottonseed | 40 |
| Rapeseed meal | 30 |
| Molasses | 22 |
| Extruded soybean | 20 |
| Mineral-vitamin premix^A^ | 40 |
| Chemical composition^B^ | |
| OM | 964 |
| CP | 172 |
| EE | 50.5 |
| NDF | 306 |
| ADF | 205 |
| Ca | 8.6 |
| P | 4.7 |
| NE_L_, MJ/kg | 7.49 |

^A^ Contained per kilogram of premix: 12 g Ca, 9 g Na, 3 g Mg, 2 g Fe, 1.75 g Zn, 1 g P, 980 mg Mn, 460 mg S, 390 mg Cu, 120 mg K, 200 000 IU vitamin A, 50 000 IU vitamin D and 1 000 IU vitamin E.

^B^ DM = dry matter. OM = organic matter; CP = crude protein; EE = ether extract; NDF = neutral detergent fiber; ADF = acid detergent fiber; NE_L_ = net energy for lactation values were estimated based on NRC (2001).

**Table S2** Summary of sequence data generated from rumen samples

| Sample | Raw reads | Clean reads |
| --- | --- | --- |
| CON_1 | 41268178 | 41153063 |
| CON_2 | 41883211 | 41780553 |
| CON_3 | 41352277 | 41225480 |
| CON_4 | 42818062 | 42671135 |
| CON_5 | 40419053 | 40282653 |
| CON_6 | 45255811 | 45083237 |
| CON_7 | 45213829 | 45049177 |
| CON_8 | 41865591 | 41737706 |
| CON_9 | 41837802 | 41725365 |
| CON_10 | 41554697 | 41390771 |
| CON_11 | 43503431 | 43365278 |
| CON_12 | 49327190 | 49216706 |
| BS_1 | 41948716 | 41838880 |
| BS_2 | 43217552 | 43057636 |
| BS_3 | 42051887 | 41935580 |
| BS_4 | 43250859 | 43156668 |
| BS_5 | 43832819 | 43691340 |
| BS_6 | 42269268 | 42134857 |
| BS_7 | 43105206 | 42906472 |
| BS_8 | 42258697 | 42133473 |
| BS_9 | 44460582 | 44344294 |
| BS_10 | 40341405 | 40216577 |
| BS_11 | 43730908 | 43618004 |
| MCE_1 | 42176538 | 42030193 |
| MCE_2 | 41617522 | 41402264 |
| MCE_3 | 41387725 | 41285883 |
| MCE_4 | 43454430 | 43228919 |
| MCE_5 | 48530727 | 48350987 |
| MCE_6 | 42576285 | 42428923 |
| MCE_7 | 45105016 | 44929219 |
| MCE_8 | 39711091 | 39495508 |
| MCE_9 | 43541500 | 43331864 |
| MCE_10 | 42968898 | 42807420 |
| MCE_11 | 51041566 | 50889031 |
| MCE_12 | 59683702 | 59420430 |
| Total | 1528562031 | 1523315546 |
| mean | 43673201 | 435233011 |
| SD | 3682844 | 3666120 |
| SEM | 622514 | 619687 |

CON = control diet; BS = control diet plus *Bacillus subtilis*; MCE = control diet plus *Macleaya cordata* extract.

| Items | Dietary treatment | | | SEM | *P*-value |
| --- | --- | --- | --- | --- | --- |
|  | CON | BS | MCE |  |  |
| Bacteria | 42.60 | 43.01 | 40.75 | 0.562 | 0.281 |
| Archaea | 0.043 | 0.040 | 0.044 | 0.001 | 0.060 |
| Eukaryota | 0.624^ab^ | 0.400^b^ | 0.945^a^ | 0.086 | 0.049 |
| Viruses | 0.045 | 0.058 | 0.040 | 0.004 | 0.557 |
| Phage | 0.045 | 0.059 | 0.043 | 0.005 | 0.603 |
| other | 56.64 | 56.44 | 58.18 | 0.491 | 0.261 |

**Table S3** Comparison of microbial domains among CON, BS or MCE cows

Data were analyzed using the Kruskal–Wallis multiple comparisons, and significance was declared at *P* value < 0.05.

CON = control diet; BS = control diet plus *Bacillus subtilis*; MCE = control diet plus *Macleaya cordata* extract.

**Table S4** The relative abundances of differential rumen bacteria and archaea among CON, BS or MCE cows

| Items | Dietary treatment | | | SEM | *P*-value |
| --- | --- | --- | --- | --- | --- |
|  | CON | BS | MCE |  |  |
| **Bacterial taxa (%)** | | | | | |
| **Phylum** |  |  |  |  |  |
| Proteobacteria | 2.34^ab^ | 2.53^a^ | 2.12^b^ | 0.105 | 0.033 |
| **Genera** |  |  |  |  |  |
| *Selenomonas* | 1.36^a^ | 1.05^b^ | 1.43^a^ | 0.047 | 0.004 |
| *Oscillibacter* | 0.62^ab^ | 0.57^b^ | 0.71^a^ | 0.023 | 0.034 |
| *Lachnospira* | 0.29^a^ | 0.30^a^ | 0.22^b^ | 0.015 | 0.030 |
| **Species** |  |  |  |  |  |
| *Firmicutes_bacterium_CAG_103* | 0.96^ab^ | 0.79^b^ | 1.28^a^ | 0.074 | 0.026 |
| *Prevotella_sp._tf2-5* | 0.85^ab^ | 0.74^b^ | 0.90^a^ | 0.030 | 0.034 |
| *Selenomonas_ruminantium* | 0.78^a^ | 0.57^b^ | 0.87^a^ | 0.034 | 0.002 |
| *Prevotella_sp._tc2-28* | 0.79^a^ | 0.61^b^ | 0.77^a^ | 0.032 | 0.040 |
| *Selenomonas_sp._AE3005* | 0.17^a^ | 0.11^b^ | 0.22^a^ | 0.012 | 0.001 |
| *Prevotella_histicola* | 0.14^b^ | 0.19^a^ | 0.14^b^ | 0.007 | 0.011 |
| *Lachnospiraceae_bacterium_AD3010* | 0.15^ab^ | 0.14^b^ | 0.17^a^ | 0.004 | 0.008 |
| *Prevotella_corporis* | 0.12^ab^ | 0.15^a^ | 0.11^b^ | 0.006 | 0.037 |
| *Corynebacterium_stationis* | 0.15^a^ | 0.07^b^ | 0.17^a^ | 0.013 | 0.001 |
| *Prevotella_disiens* | 0.12^b^ | 0.15^a^ | 0.11^b^ | 0.005 | 0.014 |
| **Archaeal taxa (%)** | | | | | |
| **Genera** |  |  |  |  |  |
| *Methanosphaera* | 5.29^a^ | 3.75^b^ | 3.40^b^ | 0.308 | 0.049 |
| *Methanobacterium* | 1.19^a^ | 1.23^a^ | 0.95^b^ | 0.047 | 0.033 |
| *Methanomassiliicoccus* | 0.43^a^ | 0.53^a^ | 0.29^b^ | 0.039 | 0.025 |
| *Methanomicrobium* | 0.38^a^ | 0.36^a^ | 0.23^b^ | 0.028 | 0.023 |
| *Methanococcus* | 0.21^a^ | 0.23^a^ | 0.14^b^ | 0.016 | 0.039 |
| *Methanoplanus* | 0.18^a^ | 0.18^a^ | 0.11^b^ | 0.016 | 0.047 |
| *Methanohalophilus* | 0.12^a^ | 0.13^a^ | 0.08^b^ | 0.010 | 0.035 |
| **Species** |  |  |  |  |  |
| *Methanosphaera_sp._WGK6* | 2.73^a^ | 1.97^b^ | 1.76^b^ | 0.128 | 0.048 |
| *Methanobrevibacter_wolinii* | 1.02^a^ | 1.11^a^ | 0.82^b^ | 0.041 | 0.040 |
| *Methanobrevibacter_sp._AbM4* | 0.72^a^ | 0.80^a^ | 0.58^b^ | 0.032 | 0.033 |
| *Candidatus_Methanomassiliicoccus_intestinalis* | 0.36^a^ | 0.47^a^ | 0.25^b^ | 0.035 | 0.032 |
| *Methanobrevibacter_cuticularis* | 0.44^a^ | 0.53^ab^ | 0.39^b^ | 0.016 | 0.012 |
| *Methanomicrobium_mobile* | 0.38^a^ | 0.36^ab^ | 0.23^b^ | 0.028 | 0.023 |
| *Methanobrevibacter_arboriphilus* | 0.47^ab^ | 0.53^a^ | 0.39^b^ | 0.020 | 0.035 |
| *Methanobrevibacter_filiformis* | 0.37^ab^ | 0.47^a^ | 0.30^b^ | 0.023 | 0.011 |
| *Methanobrevibacter_boviskoreani* | 0.29^ab^ | 0.32^a^ | 0.24^b^ | 0.010 | 0.004 |
| *Methanobrevibacter_curvatus* | 0.29^ab^ | 0.33^a^ | 0.24^b^ | 0.012 | 0.007 |
| *Methanobacterium_formicicum* | 0.23^a^ | 0.22^a^ | 0.17^b^ | 0.012 | 0.037 |
| *Methanosphaera_stadtmanae* | 0.16^a^ | 0.12^b^ | 0.11^b^ | 0.007 | 0.049 |
| *Methanobacterium_congolense* | 0.13^a^ | 0.14^a^ | 0.11^b^ | 0.005 | 0.027 |

The difference between two groups was identified by Kruskal–Wallis multiple comparisons, and the *P* value < 0.05 indicated the significant difference.

CON = control diet; BS = control diet plus *Bacillus subtilis*; MCE = control diet plus *Macleaya cordata* extract.

**Table S5** The relative abundances of protozoa in rumen of dairy cows

| Items | Dietary treatment | | | SEM | *P*-value |
| --- | --- | --- | --- | --- | --- |
|  | CON | BS | MCE |  |  |
| **Phylum** |  |  |  |  |  |
| Ciliophora | 0.428 | 0.408 | 0.462 | 0.014 | 0.156 |
| **Genera** |  |  |  |  |  |
| *Stentor* | 0.073 | 0.069 | 0.079 | 0.002 | 0.312 |
| *Oxytricha* | 0.069 | 0.065 | 0.074 | 0.002 | 0.177 |
| *Stylonychia* | 0.065 | 0.062 | 0.070 | 0.002 | 0.116 |
| *Paramecium* | 0.061 | 0.059 | 0.066 | 0.002 | 0.198 |
| *Tetrahymena* | 0.045 | 0.043 | 0.049 | 0.002 | 0.142 |
| *Ichthyophthirius* | 0.040 | 0.039 | 0.044 | 0.001 | 0.135 |
| *Pseudocohnilembus* | 0.033 | 0.031 | 0.036 | 0.001 | 0.165 |
| *Entodinium* | 0.023 | 0.024 | 0.026 | 0.001 | 0.068 |
| *Epidinium* | 0.004 | 0.003 | 0.005 | <0.001 | 0.787 |
| *Isotricha* | 0.002 | 0.002 | 0.002 | <0.001 | 0.111 |
| *Eudiplodinium* | 0.002 | 0.002 | 0.002 | <0.001 | 0.141 |
| **Species** |  |  |  |  |  |
| *Stentor_coeruleus* | 0.073 | 0.069 | 0.079 | 0.002 | 0.312 |
| *Oxytricha_trifallax* | 0.069 | 0.065 | 0.074 | 0.002 | 0.177 |
| *Stylonychia_lemnae* | 0.065 | 0.062 | 0.070 | 0.002 | 0.116 |
| *Paramecium_tetraurelia* | 0.061 | 0.058 | 0.065 | 0.002 | 0.217 |
| *Tetrahymena_thermophila* | 0.045 | 0.043 | 0.048 | 0.001 | 0.142 |
| *Ichthyophthirius_multifiliis* | 0.040 | 0.039 | 0.044 | 0.001 | 0.135 |
| *Pseudocohnilembus_persalinus* | 0.033 | 0.031 | 0.036 | 0.001 | 0.165 |
| *Entodinium_caudatum* | 0.023 | 0.024 | 0.026 | 0.001 | 0.068 |
| *Epidinium_ecaudatum* | 0.004 | 0.003 | 0.004 | <0.001 | 0.751 |
| *Eudiplodinium_maggii* | 0.002 | 0.002 | 0.002 | <0.001 | 0.141 |

The difference between two groups was identified by Kruskal–Wallis multiple comparisons, and the *P* value < 0.05 indicated the significant difference.

CON = control diet; BS = control diet plus *Bacillus subtilis*; MCE = control diet plus *Macleaya cordata* extract.

**Fig. S1** Fold changes of metabolic pathways identified in the metagenomes of the cows. CON = control diet; BS = control diet plus *Bacillus subtilis*; MCE = control diet plus *Macleaya cordata* extract.


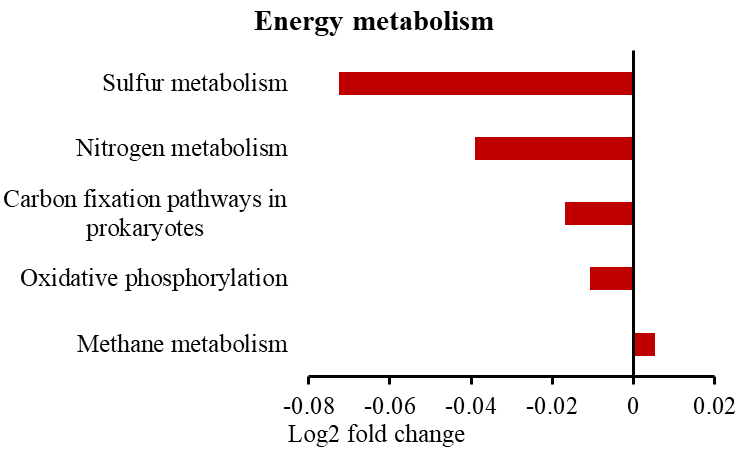

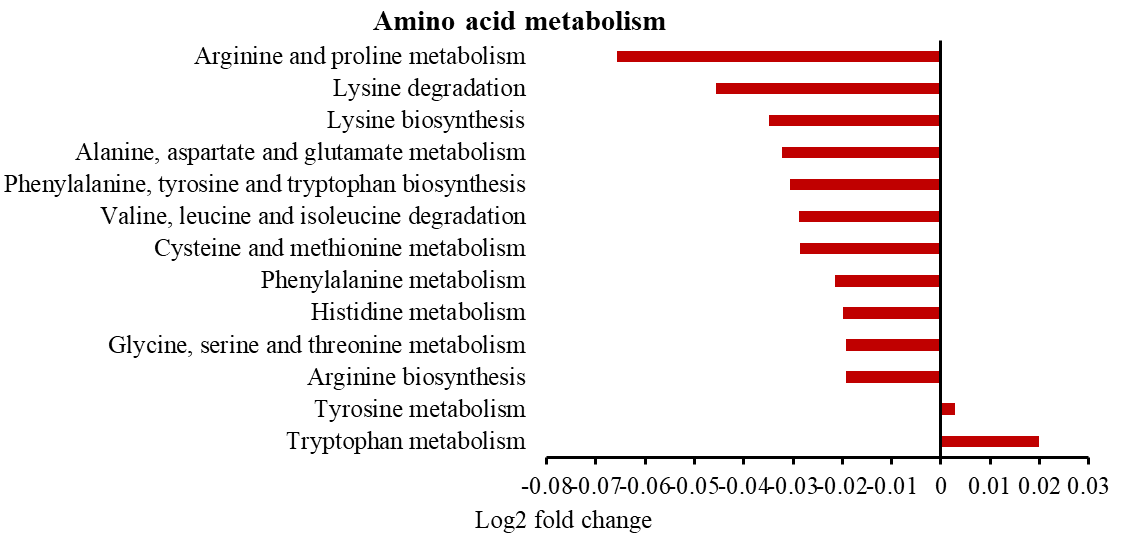

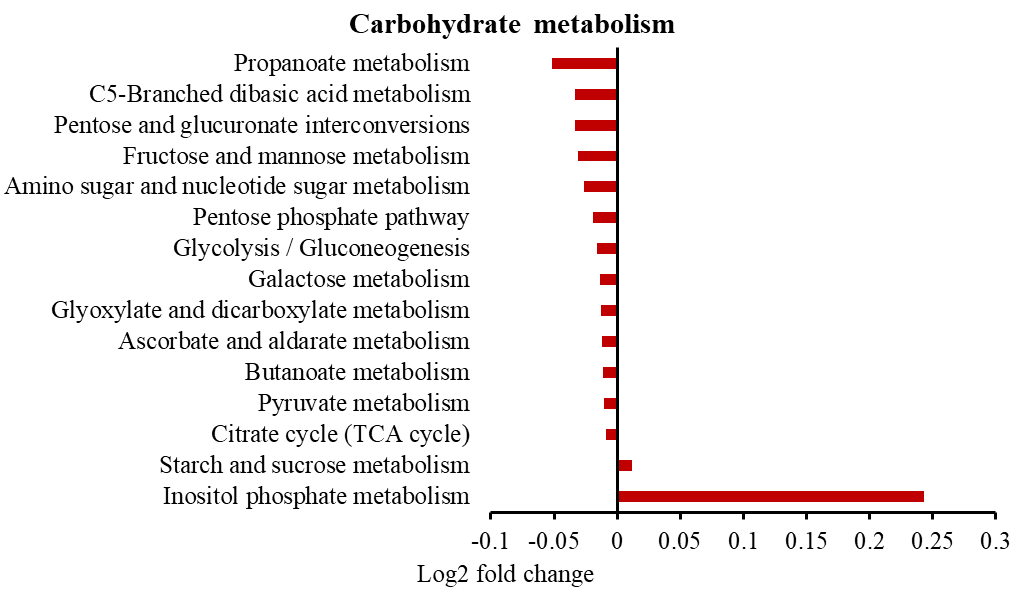

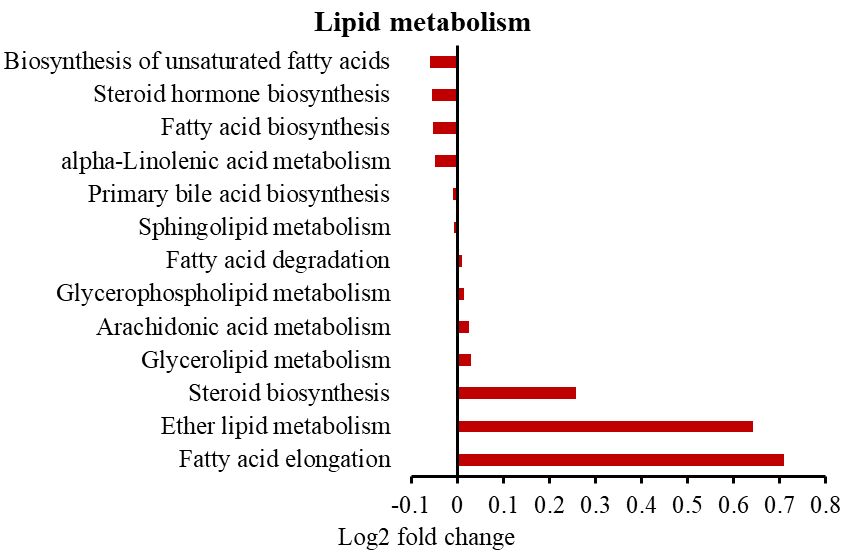


**MCE vs CON**


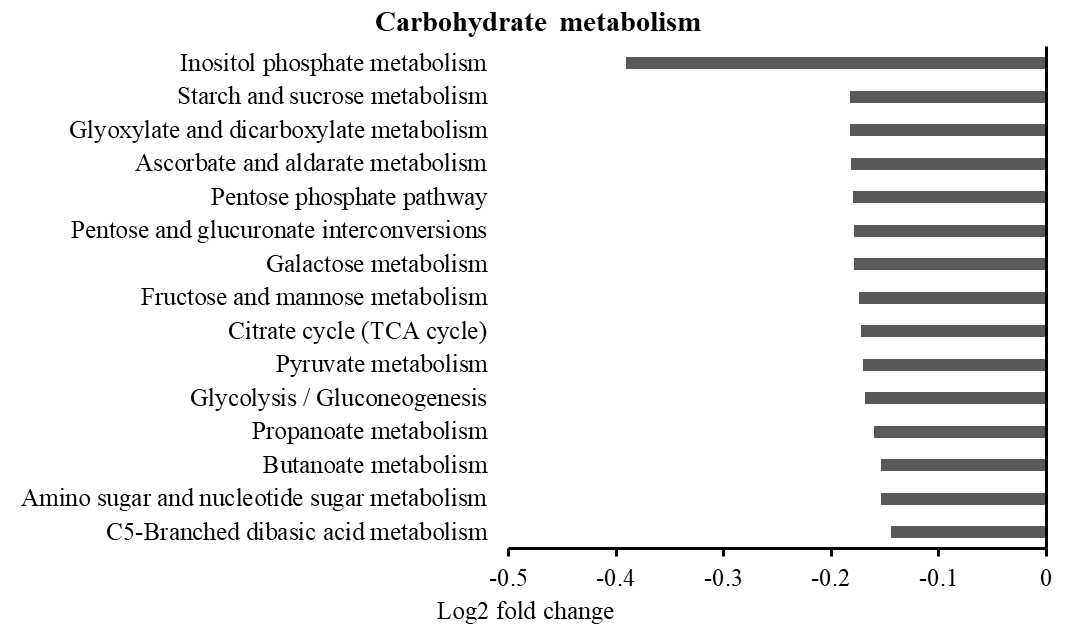

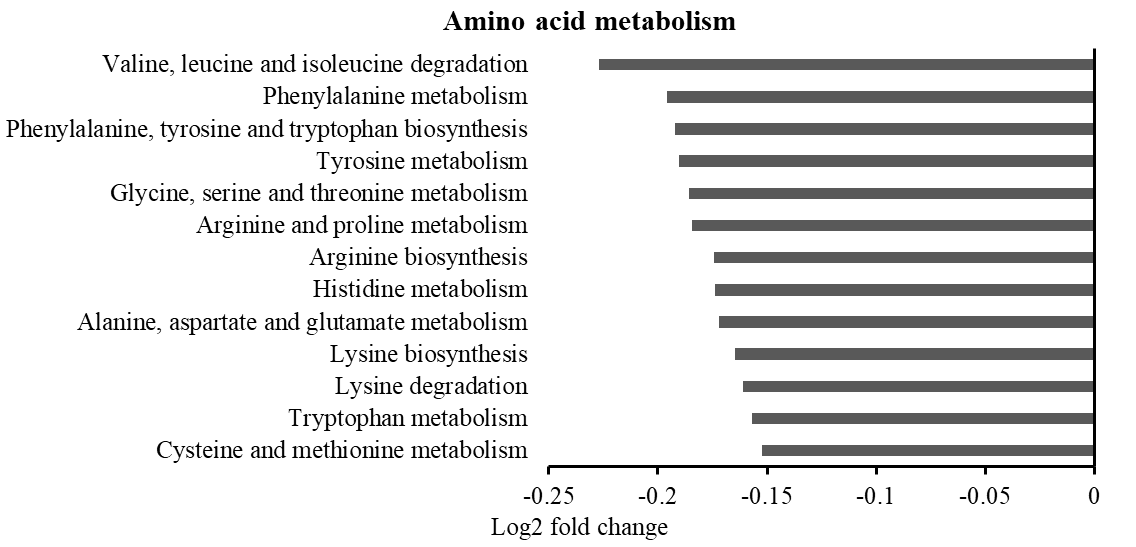

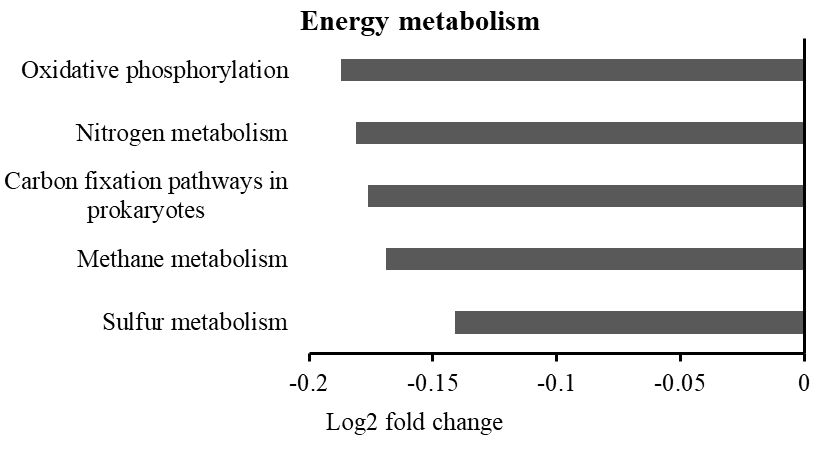

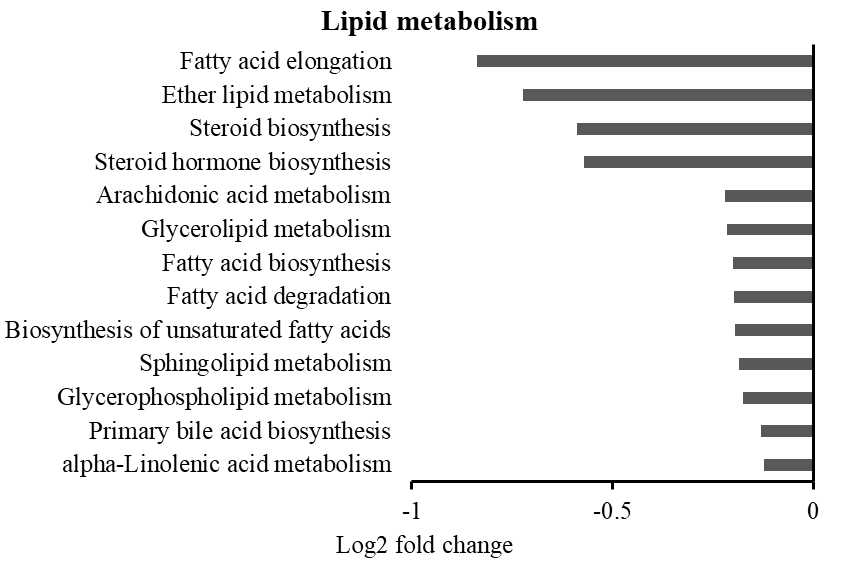


**BS vs CON**

**Table S6** The CAZymes families with significant difference in gene abundance among three groups

| CAZymes family | Dietary treatment | | | SEM | *P*-value |
| --- | --- | --- | --- | --- | --- |
|  | CON | BS | MCE |  |  |
| GH107 | 1.74^ab^ | 1.20^b^ | 2.01^a^ | 0.170 | 0.039 |
| GH119 | 0.47^ab^ | 0.35^b^ | 1.01^a^ | 0.203 | 0.037 |
| GH15 | 27.34^ab^ | 32.65^a^ | 20.66^b^ | 1.643 | 0.020 |
| GH38 | 73.45^ab^ | 68.76^b^ | 79.78^a^ | 1.881 | 0.014 |
| GH45 | 14.07^ab^ | 8.01^b^ | 21.84^a^ | 2.038 | 0.042 |
| GH71 | 0.36^a^ | 0.19^b^ | 0.27^ab^ | 0.050 | 0.029 |
| GH80 | 2.52^ab^ | 2.23^b^ | 3.18^a^ | 0.171 | 0.034 |
| GH91 | 1.32^b^ | 1.49^ab^ | 2.10^a^ | 0.145 | 0.025 |
| GH99 | 69.67^a^ | 58.81^b^ | 70.32^a^ | 2.026 | 0.037 |
| PL9 | 169.59^a^ | 142.64^b^ | 169.05^a^ | 4.665 | 0.040 |
| AA2 | 0.60^a^ | 0.60^a^ | 0.37^b^ | 0.049 | 0.046 |
| CBM18 | 0.50^ab^ | 0.19^b^ | 0.84^a^ | 0.107 | 0.025 |
| CBM57 | 26.72^ab^ | 20.47^b^ | 28.14^a^ | 1.209 | 0.030 |
| CBM62 | 48.53^ab^ | 40.96^b^ | 55.05^a^ | 1.859 | 0.015 |
| CBM8 | 12.2^ab^ | 9.56^b^ | 15.80^a^ | 0.882 | 0.019 |
| GT20 | 36.74^ab^ | 19.76^b^ | 58.08^a^ | 6.315 | 0.043 |
| GT32 | 112.67^a^ | 110.48^ab^ | 99.78^b^ | 2.027 | 0.020 |
| GT33 | 2.5^ab^ | 1.41^b^ | 3.24^a^ | 0.261 | 0.020 |
| GT53 | 2.52^a^ | 1.13^b^ | 2.65^a^ | 0.203 | 0.002 |
| GT61 | 1.1^ab^ | 0.64^b^ | 2.10^a^ | 0.201 | 0.015 |
| GT75 | 5.12^ab^ | 2.26^b^ | 9.29^a^ | 1.133 | 0.019 |
| GT82 | 0.95^a^ | 0.63^b^ | 0.68^ab^ | 0.055 | 0.044 |
| GT89 | 3.42^a^ | 2.29^b^ | 3.85^a^ | 0.176 | 0.000 |

The difference among three groups was identified by the Kruskal–Wallis multiple comparisons, and the *P* value < 0.05 indicated the significant difference.

AA auxiliary activities, CBM carbohydrate-binding modules, GH glycoside hydrolase, GT glycosyl transferase, PL polysaccharide lyases. CON = control diet; BS = control diet plus *Bacillus subtilis*; MCE = control diet plus *Macleaya cordata* extract.

**Table S7** The relative abundances of GH family genes coded fibrolytic enzymes

| CAZymes family | Dietary treatment | | | SEM | *P*-value |
| --- | --- | --- | --- | --- | --- |
|  | CON | BS | MCE |  |  |
| GH1 | 210.35 | 196.47 | 204.09 | 12.875 | 0.895 |
| GH2 | 1540.67 | 1508.88 | 1510.60 | 30.556 | 0.846 |
| GH3 | 1971.76 | 1920.94 | 1936.10 | 35.535 | 0.773 |
| GH4 | 184.64 | 173.87 | 172.59 | 3.274 | 0.261 |
| GH5 | 687.61 | 636.10 | 703.22 | 14.909 | 0.524 |
| GH6 | 3.68 | 3.29 | 4.63 | 0.253 | 0.133 |
| GH8 | 88.93 | 82.23 | 80.34 | 2.367 | 0.312 |
| GH9 | 273.10 | 267.90 | 272.58 | 7.026 | 0.980 |
| GH10 | 265.65 | 260.39 | 253.71 | 6.560 | 0.764 |
| GH11 | 18.49 | 19.51 | 19.00 | 1.023 | 0.958 |
| GH12 | 1.96 | 2.28 | 2.04 | 0.158 | 0.929 |
| GH26 | 205.77 | 200.49 | 190.30 | 5.446 | 0.468 |
| GH30 | 193.78 | 189.54 | 186.16 | 3.257 | 0.641 |
| GH31 | 950.54 | 884.73 | 944.53 | 19.004 | 0.709 |
| GH38 | 73.45^ab^ | 68.76^b^ | 79.78^a^ | 1.881 | 0.014 |
| GH39 | 91.78 | 89.00 | 91.91 | 2.012 | 0.784 |
| GH43 | 1444.68 | 1388.48 | 1374.57 | 36.624 | 0.771 |
| GH44 | 7.23 | 7.96 | 5.97 | 0.557 | 0.366 |
| GH45 | 14.07^ab^ | 8.01^b^ | 21.84^a^ | 2.038 | 0.042 |
| GH48 | 6.48 | 7.27 | 5.49 | 0.623 | 0.694 |
| GH51 | 458.48 | 446.24 | 433.29 | 12.804 | 0.607 |
| GH52 | 0.05 | 0.04 | 0.04 | 0.011 | 0.841 |
| GH54 | 20.67 | 18.61 | 21.28 | 1.728 | 0.971 |
| GH62 | 0.71 | 0.65 | 0.65 | 0.041 | 0.662 |
| GH67 | 99.48 | 93.80 | 95.75 | 2.789 | 0.726 |
| GH74 | 97.97 | 90.03 | 101.48 | 2.662 | 0.246 |
| GH92 | 394.51 | 383.32 | 372.67 | 15.439 | 0.840 |
| GH98 | 18.57 | 17.55 | 16.51 | 0.839 | 0.501 |
| GH113 | 12.17 | 12.21 | 12.73 | 0.448 | 0.525 |
| GH116 | 18.29 | 16.13 | 17.48 | 0.762 | 0.558 |
| GH120 | 72.10 | 73.19 | 72.73 | 1.916 | 0.954 |
| GH124 | 16.45 | 20.30 | 17.36 | 0.858 | 0.235 |

The difference among three groups was identified by the Kruskal–Wallis multiple comparisons, and the *P* value < 0.05 indicated the significant difference.

GH, glycoside hydrolase. CON = control diet; BS = control diet plus *Bacillus subtilis*; MCE = control diet plus *Macleaya cordata* extract.


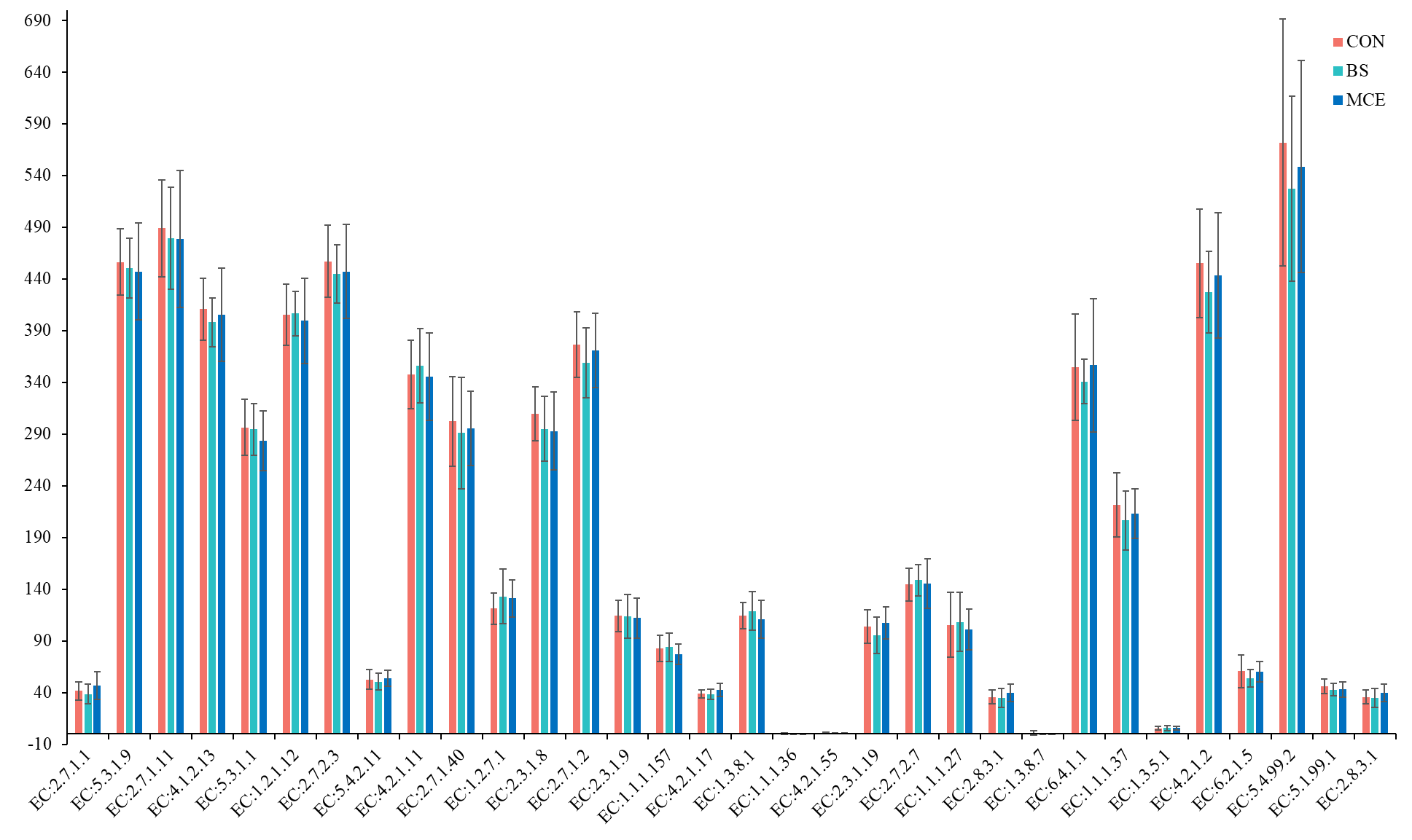
**Fig. 2** Comparisons of the abundance of KO enzymes related to the acetate, propionate, and butyrate production pathway of cows. The Kruskal–Wallis multiple comparisons was used for mean comparison, and asterisk indicated the significant difference (*P* < 0.05). CON = control diet; BS = control diet plus *Bacillus subtilis*; MCE = control diet plus *Macleaya cordata* extract.
